# A Scoping Review of Noncommunicable Disease Programs Evaluation: Bridging Gaps and Improving Frameworks in Primary Healthcare

**DOI:** 10.1002/hsr2.71444

**Published:** 2025-12-31

**Authors:** Aboalfazl Marvi, Masoumeh Sadeghi, Mohsen Aarabi, Hamidreza Shabanikiya, Fatemeh Kokabisaghi, Elaheh Houshmand, Ali Vafaee‐Najar

**Affiliations:** ^1^ Student Research Committee Mashhad University of Medical Sciences Mashhad Iran; ^2^ Department of Health Economic and Management Sciences, School of Health Mashhad University of Medical Sciences Mashhad Iran; ^3^ Department of Epidemiology, School of Health Mashhad University of Medical Sciences Mashhad Khorasan Razavi Iran; ^4^ Department of Community Medicine, School of Medicine Mazandaran University of Medical Sciences Sari Iran; ^5^ Social Determinants of Health Research Center Mashhad University of Medical Sciences Mashhad Iran

**Keywords:** monitoring and evaluation, noncommunicable diseases, primary healthcare (PHC), program evaluation, scoping review

## Abstract

**Background and Aims:**

Effective evaluation is critical for improving non‐communicable disease (NCD) programs. This scoping review explores evaluation methods at the primary healthcare (PHC) level, examines the evaluation process, maps key dimensions and indicators, and identifies related challenges.

**Materials and Methods:**

The present review was conducted based on PRISMA‐ScR and the Arksey and O'Malley framework. We systematically searched Web of Science, Scopus, PubMed, EMBASE, Emerald, Google Scholar, and relevant websites for studies published from January 2000 to January 2023. Eligible studies were selected using predefined criteria, and data were managed and synthesized using EndNote 8.1 and MAXQDA 2020.

**Results:**

A total of 50 studies were included, with most using observational studies (*n* = 22, 44%), particularly cross‐sectional designs (*n* = 17, 34%). Content analysis revealed three main categories: Evaluation processes, this category covered aspects such as evaluation levels, focus, scope, approaches, methods, and capacities. Dimension and indicators, included inputs, process, outputs, outcomes, and the perspectives of health care providers and recipients. Evaluation challenges, identified challenges included information management, workforce‐related issues, budget and deficiencies in the evaluation system itself.

**Conclusion:**

This review offers an in‐depth synthesis of evaluation methodologies applied to NCD programs within the primary healthcare context. The majority of evaluations predominantly utilized observational, cross‐sectional study designs, with a primary focused on cardiovascular diseases, diabetes, and hypertension, whereas conditions such as chronic obstructive pulmonary disease and cancer received comparatively limited attention. Evaluations mainly targeted public‐sector programs and service‐provider perspectives. In low‐ and middle‐income countries, studies often relied on external funding and evaluators. Evaluation efforts often prioritized outputs over inputs and processes and were further constrained by limited information systems, workforce shortages, budget limitations, and inadequate evaluation frameworks. Collectively, these findings emphasize the imperative for methodological diversification, inclusive engagement of a broader range of stakeholders, and enhancement of domestic evaluation capacities to establish more robust, comprehensive, and sustainable frameworks for monitoring and assessing NCD programs.

**Abbreviations**
AFRO: African Region Office
AMRO: American Region Office
CDC: Center for Disease Control and Prevention
COPD: chronic obstructive pulmonary diseases
CRCTs: clustered randomized control trial
CVD: cardiovascular diseases
DALY: disability adjusted life years
EMRO: East Mediterranean Region Office
EURO: Euroregion Office
ITS: interrupted time series
LMICs: low‐ and middle‐income countries
NCDs: noncommunicable diseases
NCDs‐GAP: Noncommunicable Diseases Global Action Plan
PHC: primary health care
RCTs: randomized control trial
SDGs: sustainable development goals
SEAR: Southeast Region Office
SW‐CRT: Stepped‐ Wedge Cluster Randomized Trial
UHC: universal health coverage
WHO: World Health Organization
WORO: Western Pacific region

## 1 Introduction

Non communicable diseases (NCDs), such as cardiovascular diseases (CVDs), cancer, chronic obstructive pulmonary disease (COPD), and diabetes, represent common risk factors for death worldwide [1]. Beyond being a health concern, NCDs pose a substantial barrier to achieving sustainable development goals (SDGs) in the 21st century [2]. In 2019, NCDs responsible for 74% of global deaths, with three‐quarters of these deaths and almost 85% of premature deaths occurring between the ages of 30 and 69 in low‐ and middle‐income countries (LMICs) [3, 4].

NCDs account for a significant health burden, representing 63.8% of disability adjusted life years (DALYs) in 2019 and impose a considerable economic burden on affected persons, families, and societies, particularly in developing countries [5]. The economic burden of NCDs is beyond the direct costs of the health care system. By reducing productivity, efficiency, and economic growth, they move countries away from the path of sustainable development [6]. It is estimated that the economic losses caused by these diseases will reach 46.7 trillion dollars by 2030 [7].

To address this challenge, the United Nations has determined a 30% reduction in premature mortality from NCDs in the SDGs. The cooperation of governments and the strengthening of the accountability of health systems are necessary to reach this goal [8]. According to recent evaluations, none of the LMICs are on the way to achieving this goal, and at least half of the countries still need to implement cost‐effective interventions to decrease the mortality rate. Evidence‐based policymaking, infrastructure enhancement, and sustainable financing are essential for the successful implementation of these interventions [9].

Given the substantial the economic burden and financial constraints associated with NCDs, policymakers must allocate limited resources effectively through evidence‐informed decision making, considering available capacities. By identifying enablers and barriers, drawing insights from past experiences, and redesigning programs [10], evaluations can provide insights to program efficiency [9, 11]. In the complex landscape of health systems involving various stakeholders, performance evaluations are crucial for informing decisions and assessing stakeholder contributions [10, 11].

Numerous studies have highlighted challenges related to resources shortages, of and infrastructure deficiencies for evaluating NCD programs [12, 13, 14, 15, 16]. More importantly, in the NCDs Global Action Plan 2013‐2030 (NCDs‐GAP), by using the phrase “What has changed?”, the World Health Organization (WHO) emphasized strengthening monitoring and evaluation [17]. Evaluation is a cornerstone for the effective management of NCD programs [16]. Moreover, developing effective programs to address NCDs requires the implementation of a well‐organized monitoring and evaluation framework [18].

The purpose of scoping reviews is to systematically identify and map the broad range of evidence available on a particular topic, field, concept, or issue, commonly regardless of the source (e.g., primary studies, reviews, etc.), within or across specific contexts. Additionally, these reviews help to pinpoint gaps in the existing literature and serve as a foundation for conducting subsequent systematic reviews [19, 20, 21]. Therefore, a scoping review was considered the most appropriate and logical type of review to provide an initial understanding of the evaluation of NCDs. One of the key phenomena in the primary healthcare system is the challenges associated with monitoring and evaluating NCD programs. Given the critical importance of this issue and the increasing volume of published research on NCD evaluation in recent years, this scoping review aimed to understand how NCD programs or interventions are evaluated in the primary healthcare setting. The specific objectives were to investigate the evaluation process and the methods used, identify dimensions and indicators employed, and recognize the challenges faced in evaluating NCD programs or interventions.

## 2 Methods

This scoping review was conducted following the PRISMA extension for scoping review (PRISMA‐ScR) (Appendix 1) [22] and the Arksey and O'Malley framework [23]. The study protocol was approved and registered on December 24, 2022, by Mashhad University of Medical Sciences, Iran (Code: IR. MUMS. FHMPM. REC.1401.156).

### 2.1 Theoretical Framework

The present study was guided by the WHO evaluation process framework [24]. This framework comprises of four‐stage [1]: Evaluation Planning: Defining evaluation questions and criteria; drafting Terms of Reference (including context, objectives, scope, focus, methodological approach, and evaluators); estimating resources; determining evaluation management structure; managing conflicts of interest; establishing a work plan; and preparing the inception report [2]. Conducting the Evaluation: Identifying information needs and data‐collection methods; briefing and supporting the evaluation team; and implementing quality assurance and control procedure [3] Reporting Results: preparing first drafts and final evaluation reports in a structured and transparent format, and [4] Utilization and Follow‑up: communication, Utilization and Follow‑up of the evaluation results.

### 2.2 Step 1. Formulation of Research Questions

This scoping review applied the PCC (Population, Concept, Context) framework [25], aligned with the aims of the study, to formulate the research questions and define the scope of the review (see Table 1).

**Table 1:** The PCC framework used in formulating the review questions.

PCC element	Description
Population (Target)	Programs and interventions targeting major non‑communicable diseases (NCDs), including **cardiovascular diseases, diabetes, cancers, and COPD.**
Concept	**Evaluation methods:** refer to the study design used for NCDs program evaluation. **Evaluation process:** encompasses selection of approaches and frameworks; determination of levels and scopes; identification of utilized resources and capacities. **Dimensions and indicators:** refer to the specific aspects and measurable indicators that are examined within the evaluation process. **Challenges in NCD program evaluation:** Barriers encountered during implementation that hinder the evaluation of NCD intervention.
Context	**Primary healthcare settings** worldwide, from January 2000 to January 2023.

1. What evaluation approaches and methods have been utilized to assess NCD programs and interventions in primary healthcare settings worldwide between January 2000 and January 2023, and what are the key components of the evaluation processes?

2. Which indicators and dimensions have been applied in evaluating NCD‐related programs within global primary healthcare contexts during January 2000 and January 2023?

3. What major challenges have been reported in the evaluation of NCD programs implemented in primary healthcare systems across different countries from January 2000 to January 2023?

### 2.3 Step 2. Search Strategy and Information Sources

A comprehensive literature search was conducted across five major databases—PubMed, Scopus, Web of Science, Embase, and Emerald Insight—as well as the Google Scholar search engine, covering the period from January 2000 (the year the Global Strategy for the Prevention and Control of NCDs was endorsed by the World Health Assembly) [26] to January 2023. The search strategy was developed and implemented based on the previously defined PCC elements. Each component—Population, Concept, and Context—was systematically translated into relevant keywords and controlled vocabularies, including Medical Subject Headings (MeSH) for PubMed and Emtree for Embase, to ensure a comprehensive and focused literature search across the selected databases. In databases lacking standardized controlled vocabularies, such as Scopus, Web of Science, Emerald Insight, and Google Scholar, keyword‐based searches were employed to complement the overall search strategy. The literature search utilized terms “noncommunicable diseases,” “evaluation,” and “primary healthcare” across the databases and the Google Scholar search engine. Keywords were combined using the Boolean operators “AND” and “OR” in all databases. Detailed search strategies for all databases are provided in Appendix 2. Additionally, the websites of the WHO, the Centers for Disease Control and Prevention (CDC), and the World Bank were manually searched. To identify further relevant studies, the reference lists of retrieved articles were also examined through hand searching. All relevant studies were imported into EndNote software (version 8.1) for management.

### 2.4 Step 3. Eligibility Criteria and Study Selection

Eligibility criteria were established in alignment with the study objectives. The inclusion and exclusion criteria for study selection are presented in Table 2. To ensure clarity and consistency, the criteria were initially piloted on a random sample of ten studies and refined based on the feedback obtained. Following the removal of duplicates, titles and abstracts were independently screened by two reviewers. Nonrelevant records were excluded, and the full texts of the remaining studies were independently assessed for eligibility by both reviewers. Any discrepancies during the screening process were resolved through discussion and consensus with the research team.

**Table 2:** Inclusion and exclusion criteria.

No	Item	Inclusion criteria	Exclusion criteria
1	Publication type	The original articles and relevant reports on program evaluation.	Review articles, conference and seminar papers, commentaries, theses, letters to the editor, study protocols, and irrelevant evaluation reports.
2	Study design	All study designs that reported NCD program evaluation.	—
3	Study focus	Studies that evaluated prominent NCD programs (including Cardiovascular, Diabetes, COPD, and Cancers).	Studies or reports outside the study focus.
4	Study level and scale	Primary health care level and any scale (country, state, cities, population, and individual level).	Studies or reports outside the study level.
5	Language	English	Papers in other language.
6	Date	Studies published in the period between 1 January 2000 to January 2023.	Studies or reports published outside the specified period.
7	Access, incomplete texts, duplicates	Full text available.	Lack of access to the text of articles or reports, incomplete text or Duplications.

### 2.5 Step 4. Data Extraction and Charting

A data extraction form was developed in Microsoft Excel and initially piloted on a sample of five studies. Revisions were made based on feedback from the research team (See Appendix 7). Two independent reviewers systematically extracted data from all included studies, and any discrepancies were resolved through discussion or by consulting a third reviewer. To ensure consistency and accuracy, one researcher reviewed and consolidated the extracted data into a single format for analysis. Although a formal quality assessment was not performed—consistent with scoping review methodology [27]—the research team reviewed the data for completeness and accuracy. Extracted information included study characteristics (e.g., authors, study design, country, WHO region, and World Bank income level) as well as key findings relevant to the research questions. The results were then categorized in alignment with the study objectives.

### 2.6 Step 5. Data Synthesis and Reporting

Following data extraction, we systematically organized and synthesized the collected data to address the research questions. To answer the first research question, we analyzed data using the Framework Method—a structured technique for identifying key themes and patterns [28]. guided by the WHO evaluation framework. We synthesized results under a main category, “Evaluation Process,” derived from this framework, and then applied six subcategories—evaluation level, focus and scope, approach, methods, framework, and capacity—because they aligned closely with our study objectives. We excluded other framework components that fell outside the scope of this review. In this phase, we first conducted open coding to identify key concepts. We then grouped similar and related codes under the predefined subcategories, resulting in a structured and deductive synthesis of the data. To answer the second research question, we categorized the dimensions and indicators used to evaluate non‐communicable disease programs according to the classification presented in the reviewed articles, within the framework of a logic model (inputs, processes/activities, outputs, and outcomes) [24]. In Step 3, to identify challenges in evaluating NCD programs, we employed Braun & Clarke's thematic analysis [29] using an inductive approach. We began by thoroughly reviewing the texts of the selected studies and extracting initial codes, which included identified challenges. These initial codes were iteratively refined and organized into sub‐themes, which were then synthesized into higher‐level main themes. Finally, all main themes were consolidated under a single overarching category labeled “Challenges.” We implement a structured coding process using MAXQDA 2020, integrating both deductive and inductive approaches. Three researchers participated in the coding process: two (EH and AM) independently coded the data, while the third (MA) provided oversight. Discrepancies were resolved through consensus or consultation with the research team. The entire process was supervised to ensure consistency and rigor. For a detailed overview of the coding process, please refer to Appendices 3 and 8.

### 2.7 Rationale for Excluding Quality Assessment

Although formal quality appraisal is typically part of systematic reviews, it was not conducted in this scoping review. According to updated methodological guidance for the conduct of scoping reviews [27]. the primary purpose of this scoping review is to map existing evidence, rather than to critically appraise study quality. Additionally, the included studies exhibited substantial heterogeneity in design, methodology, and reporting quality, rendering a formal quality assessment both challenging and unlikely to add meaningful value. Consequently, our focus remained on synthesizing and categorizing evaluation processes, approaches, indicators, and identified challenges, rather than judging the methodological rigor of each study.

## 3 Results

### 3.1 Study Identification and Selection

**Figure 1:**
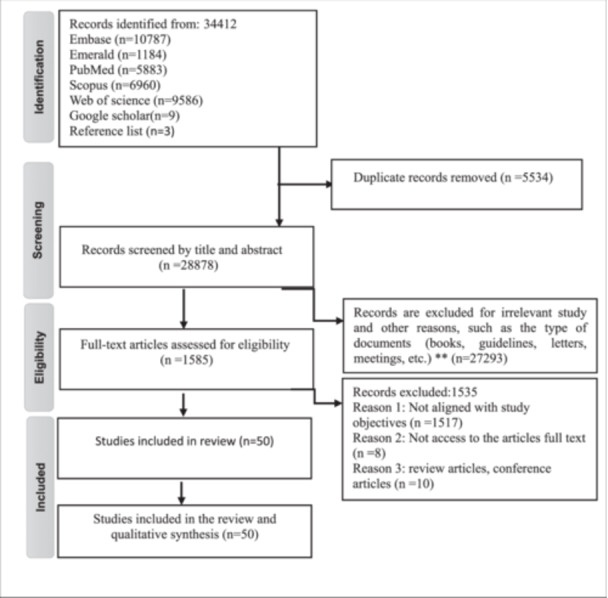
PRISMA flow diagram of study selection.

In total, 34,412 records were identified, with 34,400 from database searches and 12 from other sources. Following title and abstract screening, 1585 articles were assessed in full text. Of these, 50 studies [30, 31, 32, 33, 34, 35, 36, 37, 38, 39, 40, 41, 42, 43, 44, 45, 46, 47, 48, 49, 50, 51, 52, 53, 54, 55, 56, 57, 58, 59, 60, 61, 62, 63, 64, 65, 66, 67, 68, 69, 70, 71, 72, 73, 74, 75, 76, 77, 78, 79]. fulfilled the inclusion criteria and were included in the analysis. The PRISMA‐ScR flow diagram summarizes the selection process (Figure 1).

### 3.2 Publication Timeline and Geographic Distribution

**Figure 2:**
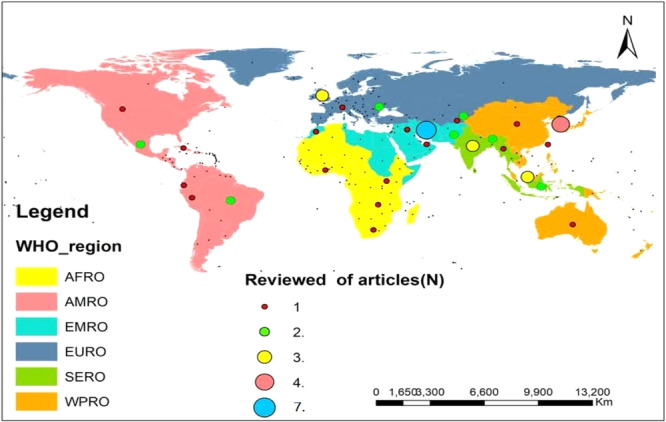
Geographic distribution of included studies by WHO regions.

**Figure 3:**
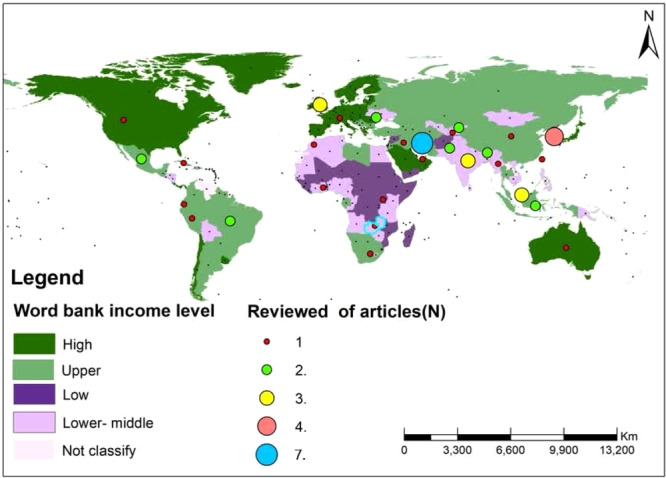
Geographic distribution of included studies by World Bank income level.

This review covered studies published from 2006 to 2023, the majority of which (38 studies, 76%) appeared after 2017 (Appendix 4). The geographic and economic distribution of the studies was illustrated through GIS‐based mapping by WHO region [80]. and World Bank income levels [81]. (Figures 2 and 3). These maps showed that most research was conducted in the Eastern Mediterranean Region and in upper‐income countries. Frequencies and percentages for all categories are summarized in Table 3.

**Table 3:** Frequency and percentage of studies by WHO region and income level.

Category/WHO Region/Income level	No. of Studies/%
**WHO Regions**	
Eastern Mediterranean Region (EMRO)	12 (24.48%)
Western Pacific Region (WPRO)	11 (22.44%)
European Region (EURO)	9 (18.36%)
Region of the Americas (AMRO)	7 (14.28%)
South‐East Asia (SEARO)	7 (14.28%)
African Region (AFRO)	3 (6.12%)
One study was conducted across multiple WHO regions	
**World Bank Income Levels**	
Upper‐middle‐income countries	22 (45.83%)
Lower‐middle‐income countries	13 (27.08%)
High‐income countries	11 (22.91%)
Low‐income countries	2 (4.16%)
Two studies were conducted across multiple income‐level	

### 3.3 Study Design Characteristics

Among the 50 studies included in this review, 48 (96%) were original research articles and 2 (4%) were evaluation reports. The studies encompassed observational, mixed‐methods, interventional, and qualitative designs. Detailed study types are presented in Table 4, while the proportional distribution of study designs is shown in Table 5. Among the included studies, cross‐sectional designs were the most frequently reported.

**Table 4:** Characteristics and key findings of the included studies.

References	Study Design	Country/WHO regions (World Bank income Level)	Study setting (Evaluation level)	Program scale (Evaluation focus & scope)	Evaluation Approach	Evaluation method	Evaluation farmwork	Evaluation Capacity Funder, (Evaluators)
Husin, M, 2023 [30]	Before‐after	Malaysia/WPRO (Upper)	Public PHC clinics (Regions, Micro)	Pilot (Diabetes)	OE	Quantitative	Theoretical model based Donabedian& TRIAD model	Internal (Internal)
Partovi, Y, 2023 [31]	Mixed‐ methods	Iran/EMRO/(Upper)	National level	Program (NCDs program)	PE	Mixed‐Methods	PAST	Internal (participatory)
Kumar, s, 2022 [32]	Cross‐ sectional	India/SEARO (Lower‐middle)	PHC centers (States, Meso)	Pilot (Brest cancer)	PE & OE	Quantitative	—	External (participatory)
Tenzin, K, 2022 [33]	Mixed ‐methods	Bhutan/SEARO (Lower‐middle)	Hospital & basic health units (districts, Micro)	Pilot (Hypertension and Diabetes)	PE & OE	Mixed‐methods	—	External (Participatory)
Ayat, S, A, 2022 [34]	Retrospective cohort	Iran/EMRO (Upper)	General hospital& PHC centers (City, Micro)	Pilot (CVD)	OE	Quantitative	—	Internal (Internal)
Kim, H, S, 2022 [35]	Before‐after	South Korea/WPRO (High)	PHC Clinics (cites, Micro)	Pilot (Hypertension and Diabetes)	OE	Quantitative	Logic model	Internal (Internal)
Shafiee, G, 2022 [36]	Cross‐ sectional	Iran/EMRO (Upper)	Urban and rural area (PHC centers) (national)	Program (Diabetes)	OE	Quantitative	—	Internal (Internal)
Lee, E, W, 2022 [37]	Before‐after	South Korea/WPRO (High)	PHC clinics (cites, Micro)	Pilot (hypertension)	OE	Quantitative	Logic model	Internal (Internal)
Shanmuganathan S.2022 [38]	Mixed methods	Malaysia/WPRO (Upper)	National level	Program (CVD, Diabetes, Cancers)	PE	Mixed‐ methods	PAST	‐(Participatory)
Patel, R, 2020 [39]	Cross‐sectional	England/EURO/(High)	90% Family physician centers (national)	Program (CVD Risk score and Risk factors, Diabetes, Hypertension, Hyperlipidemia)	PE	Quantitative	—	Internal (Internal)
“Izadi, S, 2021 [40]	Cross‐sectional	Iran/EMRO (Upper)	PHC Centers (cites, Micro)	Pilot (Cervical Cancer screening)	OE	Quantitative	—	‐(Internal)
Low, LL, 2021 [41]	Mixed methods	Malaysia/WPRO (Upper)	Public PHC clinics (region, Micro)	Pilot (Diabetes, Hyper lipidemia and hypertension	PE	Mixed‐ methods	—	Internal (Internal)
Moradi, G. et al. 2021 [42]	Cross sectional	Iran/EMRO (Upper)	Community (Provinces, Meso)	Program (Diabetes)	OE	Quantitative	—	Internal (Internal)
Laatikainen, T. et al. 2020 [43]	Cluster randomized control trial	Moldova/EURO (Upper)	PHC centers (Regions, Micro)	Pilot (CVD risk score and Risk factors screening, Hypertension & diabetes)	PE & OE	Quantitative	—	External (Participatory)
Collins, D. et al. 2020 [44]	Cluster randomized control trial	Moldova/EURO (Upper)	PHC centers (Regions, Micro)	Pilot (CVD risk score and Risk factors screening, Hypertension and diabetes management)	PE & OE	Quantitative	—	External (Participatory)
Aye, L. L. et al. 2020 [45]	Mixed‐methods	Myanmar/SEARO (Lower‐middle)	PHC centers (Town ship, Micro)	Pilot (NCDs screening, CVD risk Score and Risk factors, breast and cervix cancer, Diabetes & Hypertension)	PE & OE	Mixed‐methods	—	External (Participatory)
Gonzalez, Y. V. et al. 2020 [46]	Cross‐sectional	Cuba/AMRO (Upper)	Polyclinic (Provinces/State, Micro)	Pilot (hypertension)	OE	Quantitative	PAHO ‐ PEN HEART	Participatory (Participatory)
Flor, L. S. et al. 2020 [47]	Mixed‐methods	Brazil, India, South Africa and the USA/AMRO, SEARO, AFRO, AMRO (Upper, lower‐middle, upper, high)	Community level (regions, Meso)	Pilot (Risk factors, Diabetes and hypertension)	PE & OE	Mixed‐methods	Logic model	External (External)
Miranda‐Machado, P. et al. 2020 [48]	Retrospective cohort study	Colombia/AMRO (Upper)	Public‐ private, Community level (region, Meso)	Program (CVD)	OE	Quantitative	—	‐(Internal)
Lim, S. M. et al. 2020 [49]	Retrospective cohort study	South Korea/WPRO (High)	PHC clinics (cites, Micro)	Pilot, (Hypertension and Diabetes)	OE	Quantitative	—	Internal (Internal)
Teh, X. R. et al. 2020 [50]	Cross‐sectional	Malaysia/WPRO (Upper)	PHC clinics (districts, Micro)	Pilot (Hypertension)	OE	Quantitative	—	Internal (Internal)
Barrera‐Guarderas, F. et al. 2020 [51]	Prospective cohort study	Ecuador/AMRO (Upper)	Community healthcare centers (districts, Micro)	Program (Diabetes)	OE	Quantitative	—	‐(Internal)
Jong Koo Kim. Et al. 2020 [52]	Before‐and‐after	Peru/AMRO (Upper)	PHC centers (districts, Micro)	Pilot (Hypertension)	OE	Quantitative	—	External (Participatory)
Collins, D. R. J. et al. 2019 [53]	Cluster randomized control trial	Tajikistan/EURO (Lower‐middle)	PHC centers (District, Micro)	Pilot (Risk factors, CVD risk score, hypertension management)	PE & OE	Quantitative	—	External (Participatory)
Birabwa, C. et al. 2019 [54]	Mixed methods	Uganda/AFRO (low)	Hospitals and Rural Health Centers (district, Micro)	Program (Diabetes)	PE & OE	Mixed‐methods	Kruk model	Internal(Internal)
Adler AJ. Et al.2019 [55]	Prospective cohort	Ghana/AFRO (Lower‐middle)	Public private, Peri‐urban community (District, Micro)	Pilot (Hypertension)	OE	Quantitative	—	External (Participatory)
Peiris, D, 2019 [56]	Mixed‐methods	Australia/WPRO/(High)	Community (state, meso)	Pilot (CVD, Diabetes, hypertension)	PE & OE	Mixed‐Methods	Logic model	‐(Internal)
Jayanna, K, 2019 [57]	Mixed‐methods	India/SERO/(Lower‐middle)	Public ‐private community center (City‐Micro)	Pilot (Diabetes, Hypertension)	PE & OE	Mixed‐methods	—	External (Participatory)
Kadhim Yasir, M, 2018 [58]	Mixed‐methods	Iraq/EMRO/(Upper)	PHC centers (city, micro)	Pilot (Asthma)	PE & OE	Mixed‐methods	—	‐(Internal)
Khan, M. A. et al. 2018 [59]	Mixed‐methods	Pakistan/EMRO (Lower‐middle)	Private PHC clinics (City, Micro)	Pilot (Hypertension)	PE	Mixed‐methods	Logic model	External (Participatory)
Khan, M. A. et al. 2018 [60]	Mixed‐methods	Pakistan/EMRO (Lower‐middle)	PHC Centers (province/state, Micro)	Pilot (Diabetes)	PE	Mixed‐methods	Logic model	External (Participatory)
Duan, K. et al. 2018 [61]	Before‐ after	Mexico/AMRO (Upper)	PHC clinics (Region, Micro)	Pilot (Diabetes& hypertension)	PE & OE	Quantitative	—	External (Participatory)
Yan, L. D. et al. 2017 [62]	Mixed – methods	Zambia/AFRO (Lower‐ middle)	Rural PHC clinics (district, Meso)	Program (Hypertension)	PE & OE	Mixed‐methods	—	External (Participatory)
Hyon, C. S. et al. 2017 [63]	Cross‐sectional	North Korea/SEARO(low)	Polyclinics in (City, Micro)	Pilot (Risk factors screening, CVD risk score, risk factors, Diabetes and hypertension)	PE & OE	Quantitative	—	External (Participatory)
Simão, C. C. A. L. et al. 2017 [64]	Cross‐sectional	Brazil/AMRO (Upper)	PHC units (district, Micro)	Program (Diabetes)	PE & OE	Quantitative	Donabedian	External (Internal)
Tania, C. et al. 2017 [65]	Mixed methods	Switzerland/EURO (High)	Community level (Region, Micro)	Pilot (COPD)	PE & OE	Mixed‐methods	—	External (Internal)
Kontsevaya A, Farrington J. 2017 [66]	Before‐after	Kyrgyzstan/EURO (Lower‐middle)	20 Family health centers (city, Micro)	Pilot (NCDs screening: Risk score, risk factor, CVD, diabetes and hypertension)	OE	Quantitative	—	External (External)
Collins. D, et al. 2017 [67]	Qualitative	Kyrgyzstan/EURO (Lower‐middle)	20 Family health centers (city, Micro)	Pilot (Risk score, risk factor, Diabetes, Hypertension, CVD,)	PE	Qualitative	—	External (External)
“Robson, J. et al. 2016 [68]	Cross‐sectional	England/EURO (High)	655 Family medicine centers (national)	Program, (CVD Risk score and Risk factors, Diabetes, Hypertension, Chronic Kidney Diseases)	OE	Quantitative	—	Internal (Internal)
El Fakir, S. et al. 2015 [69]	Cross‐sectional	Morocco/EMRO (Lower‐middle)	PHC facility (city, Micro)	Pilot (Breast cancer)	OE	Quantitative	—	Internal (Internal)
Kim Y, et al. 2015 [70]	Before and after	South Korea/WPRO (High)	PHC Clinic of National Health Insurance (Regions, Micro)	Program (Hypertension)	OE	Quantitative	—	Internal (Internal)
Backer, C, 2015 [71]	Cross‐ sectional	England/EURO/(High)	83 Family Physician centers (city, micro)	Program (Diabetes, hypertension, risk factors)	PE	Quantitative	—	Internal (Internal)
Yeh, Y. P. et al. 2014 [72]	Cross sectional	Taiwan/WPRO (High)	Hospitals & 27PHC clinics (cites, Micro)	Program (Diabetes)	PE, OE	Quantitative	RE‐AIM model	Internal (Internal)
Liang, X. H. et al. 2014 [73]	Cross sectional	China/WPRO (Upper)	10 communities (district, Meso)	Program (Hypertension and Diabetes)	OE	Quantitative	—	Internal (Internal)
Wangchuk, D. et al. 2014 [74]	Before‐ After	Bhutan/SEARO (Lower‐middle)	7 Health centers, 2 hospitals (districts, Micro)	Pilot (CVD risk score, COPD, Risk factors, Hypertension and Diabetes)	OE	Quantitative	—	External (Participatory)
Farzadfar F, et al. 2012 [75]	Cross sectional	Iran/EMRO (Upper)	Community (national)	Program (Diabetes and hypertension)	OE	Quantitative	—	‐(Internal)
López‐López E. et al. 2012 [76]	Cross‐sectional	Mexico/AMRO(Upper)	40 PHC Clinics (Region, Meso)	Program (Diabetes)	OE	Quantitative	Effective coverage	Internal (Internal)
Krishnan, A. et al. 2011 [77]	Mixed‐methods	India and Indonesia/(SEARO) (Lower middle, Upper)	Urban low‐income communities (Cities, Meso)	Pilot, (Risk factors screening and management)	FE, OE	Mixed‐methods	CINDI	External (Participatory)
Baynouna, L, 2010 [78]	Before‐ after	United Arab Emirates/EMRO/(high)	PHC centers (city‐ micro)	Pilot (Diabetes, Hypertension, risk factors)	OE	Quantitative	—	‐(Internal)
Sarrafzadegan, N. et al. 2006 [79]	Cross sectional	Iran/EMRO (Upper)	Community level,(cites, Meso)	Program,(CVD Risk factors screening and management)	OE	Quantitative	IHHP model bases on logic model	External(Participatory)

*Note:* 1. WHO region: EMRO: East Mediterranean Region Office; AFRO: African Region Office; EURO: Euroregion Office; AMRO: American Region Office; SEAR: Southeast Region Office; WPRO: Western Pacific region.

2. The evaluation levels have been categorized according to the study′s location including micro level (city or region), meso level (province/state) and macro level (national).

3. NCDs: Non‐communicable Diseases; CVD: Cardiovascular Diseases; COPD: Chronic Obstructive Pulmonary Diseases;

4. PE: Process evaluation; OE: Outcome evaluation (effectiveness); FE: Formative evaluation

5. IHHP model: Isfahan health heart program; CINDI Framework: Integrated non‐ communicable disease intervention; RE‐AIM model: reach, effectiveness, adaptation, implementation, maintenance; effective coverage: mentioned in the discussion section; logic model: a logic model is a framework that demonstrate the relationship between a program′s inputs, processes and outputs; Donabedian framework: the Donabedian framework is used for health care quality assessment, according to the model the quality can be evaluated based on three dimension included structure, process and outcome, PAST: The program sustainability assessment tool, TRIAD: Translating research into action for diabetes model. Theoretical framework: Facility factors, Patients factors, healthcare provider factors, process of care, patients′ behavior, clinical outcome.

**Table 5:** Study designs of included studies.

Study design	No. of studies/%	Subtypes/Notes
Observational	22 (44%)	− Cross‐sectional: 17 (77.27%) − Prospective cohort: 2 (9.09%) − Retrospective cohort: 3 (13.63%)
Mixed‐methods	15 (30%)	—
Interventional	12 (24%)	− Non‑randomized controlled trials (NRTCs) (pre–post): 9 (75%) − Randomized controlled trials (RCTs): 3 (25%)
Qualitative	1 (2%)	—

### 3.4 Three‑Step Analytical Framework

A content analysis was conducted on the included studies, based on the theoretical frameworks described in the Methods. Analyses followed three systematic steps: mapping evaluation processes, approaches, and methods to the WHO Evaluation Process Framework; extracting indicators via the framework's logic model; and identifying challenges using Braun & Clarke's thematic analysis. The findings are presented according to this structured approach.

### 3.5 Evaluation Process

The analysis of studies included in this review highlighted key elements of evaluation processes, approaches, and methods. Findings were systematically mapped to the WHO Evaluation Process Framework. The primary category, “Evaluation Process,” comprised six subcategories: evaluation level, focus, scope, approaches, methods, frameworks, and existing capacities. Table 4 presents the details, and Table 6 shows the categories and subcategories.

**Table 6:** Key findings related to the evaluation process of NCD program.

WHO Evaluation process framework (Sub categories)	Findings
Evaluation Level	− Most studies 34 (68%) were conducted at the micro (cites) level. − 10 (20%), and 6 (12%) of studies were conducted at meso (province/states), and macro levels(national), respectively. − 38 (76%) of evaluations were center‐based, while 10 (20%) were community‐based. − Only two studies (4%) assessed NCD programs at the policy or managerial levels. See Table 4.
Evaluation focus and scope	− 32 (64%) of the evaluated programs were in the pilot phase. − CVD, hypertension, diabetes, and their common risk factors were emphasized in 45 (90%) of studies. − Cancers were evaluated in 5 studies (10%), and COPD in 3 studies (6%). − 46 (92%) of studies focused on programs implemented in the public sector. − Only 4 (8%) of studies evaluated programs executed through public–private partnerships or the private sector. See Table 4.
Evaluation approach	− Four main approaches were used in NCD programs evaluation: outcome‐based 24 (48%), process‐based 8 (16%), formative–outcome (used in only one study), and process–outcome 17 (34%). See Table 4. − Formative evaluation approach, assesses the feasibility, acceptability, and suitability of a program before its full implementation. − The process approach evaluated NCD program in terms of structure (e.g., Services provision, readiness, organization capacity), process (e.g., Standard and guideline‐based services delivery, referral system, coordination, patients and staff training, acceptance, services registry and data quality), and effects on stakeholders (e.g., patients and services provider satisfaction, facilitators, barriers, effectiveness and implementation costs), and the role of socioeconomic context (e.g., sustainability and environment support) in the success of programs in different dimensions. − In 11 (44%) of the studies, feasibility was the most evaluated indictor, see implementation outcome in see Table 4 and Appendix 4. − The outcome‐based approach evaluated the NCD program in terms of effectiveness (e.g., clinical outcome). See Appendix 4.
Evaluation methods	− In general, 34 (68%) of studies used quantitative methods, 15 (30%) mixed methods and 1 (2%) qualitative method. See Table 3. − (30%) studies examined managers/policymakers, service providers, and patient perspectives. − Provider viewpoints were most prominent 6 (40%), while patient perspectives were captured in only one study (6.7%) and manager perspectives in two (13.3%). Comprehensive stakeholder integration—including all three groups—was present in just one study (6.7%), and combined patient–provider viewpoints appeared in five studies (33.3%). See Appendix 6. − In terms of methodology, 22 studies (44%) mentioned small sample size, sampling method, and lack of a control group as research limitation. − In addition, interventional studies also face limitations including unmet parallel trends assumptions, misestimations due to data quality issues, neglect of side effects, and time constraints.
Evaluation framework	− Framework usage: Overall, 16 studies (32%) employed a formal model or framework for evaluating NCD programs. − Framework type: Logic model: used in 6 studies, PAST: used in 2 studies, RE‐AIM, Donabedian, Kruk, PAHO‑PEN HEART, CINDI, Effective Coverage, IHHP‐based Logic model, Theoretical‐model‐based Donabedian, TRIAD, and Cognitive Theory: each applied in 1 study. See Table 4.
Evaluation capacity	− Funding sources: overall, 19 studies (38%) were funded by domestic research institutions and organizations; 22 studies (44%) received funding from international or charitable organizations, or through international cooperation; one study had mixed domestic–international funding; and eight studies (16%) did not report their funder. − Geographic & funding context: in LMICs, 13 evaluation studies (86.6%) were conducted with international funding, and two studies were supported by internal funders. − In upper‐middle‐income countries, among 22 reviewed studies, 9 (40.9%) were funded by domestic sources, 6 (27.27%) by international funders, and 1 study had joint funding. Funding information was not reported in 6 studies. − In high‐income countries, 8 out of 11 studies (72.72%) were funded by domestic sources, 1 study was supported by international funding, and 2 studies did not report their funding sources. − Evaluator capacity: overall, internal evaluators and researchers conducted 27 studies (54%); external evaluators conducted 3 studies (6%); and 20 studies (40%) were conducted through collaboration between internal and external evaluators. − Evaluator capacity: In LMICs, 11 studies (73%) conducted with international evaluators and researchers participated; two studies were led solely by external evaluators, and two by internal evaluators or researchers. − In Upper – middle income, 14 studies (63.63%) were assessed by domestic evaluators, while 8 studies (36.36%) involved collaborative evaluation − In high‐ income countries, all studies (100%) were evaluated by domestic researchers or evaluators. See Table 4.

### 3.6 Dimensions and Indicators

**Figure 4:**
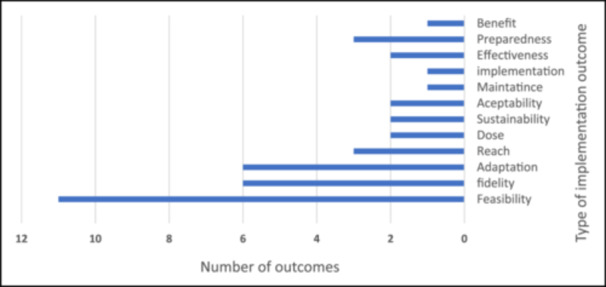
Frequency of reported implementation outcomes across the included studies.

The included studies were systematically analyzed to identify dimensions and indicators used in NCD program evaluations, categorized into inputs, processes, outputs, and outcomes. This classification aligns with the Logical Framework Model [24] and is detailed in Appendix 5. Table 7 summarizes the main dimensions and the number of studies reporting each indicator. Among implementation outcomes, feasibility, fidelity, and adaptation were most frequently reported, reflecting a focus on initial program implementation, particularly in pilot programs (see Figure 4). Clinical outcomes primarily focused on managing diabetes and hypertension, with most indicators capturing short‐ and midterm effects. Full details of all indicators are provided in Appendix 5.

**Table 7:** Dimensions and indicators in NCD program evaluation.

Category	Indicators	No. of studies/%
Inputs	– Human resources (availability and accessibility of personnel)– Equipment (availability of basic devices)– Physical infrastructures– Organizational factors (care delivery and referral systems)– Medicines and diagnostic supplies (availability and access to essential medications and diagnostic tools)	6 studies (12%)
Processes	– Service delivery: prevention, screening, diagnosis, treatment, referral, follow‐up– Service availability– Service recording and record keeping– Training for patients and providers	16 studies (32%)
Outputs	– Number of patients diagnosed, receiving care, under active treatment– Measures of patient engagement– Number of patients referred and followed up– Number/percentage of patient records initiated and completed– Count of trained staff– Percentage of staff supervision	24 studies (48%)
Outcomes	**Implementation Outcomes:**– Feasibility– Fidelity– Adaptation– Reach– Dose– Sustainability– Acceptability– Implementation– Maintenance– Effectiveness– Preparedness– Benefit	17 studies (34%)
	**Other outcomes:** – Patient/staff awareness– Treatment costs– Physician–patient relationships– Staff knowledge and attitudes– Staff performance– Service utilization– Satisfaction– Access to healthcare	11 studies (22%)
	**Clinical outcomes:**– In CVD, Hypertension & Diabetes programs: blood pressure control, glycemic control, adherence to treatment and care, improvement in behavioral risk factors, reduction in cardiovascular risk score, disease complications, mortality, hospitalization rate– In cancer programs: screening coverage and diagnosis rates– In Asthma and COPD programs: symptom reduction, disease control, and quality of life improvements	38 studies (76%)

Additional findings included insights from stakeholders, which were identified as both strengths and weaknesses. Strengths were mainly observed in improved service delivery, while weaknesses were found in areas such as intersectoral collaboration, financing, workforce challenges, information systems, and the availability of medicines and equipment (see Appendix 6).

### 3.7 Challenges

Challenges in evaluating NCD programs were categorized into four main themes: information management, workforce issues, budget constraints, and evaluation systems, comprising 13 subthemes (Table 8).

**Table 8:** Challenges facing the evaluation of NCD programs.

Thems	Subthemes	Reference
**Information management**	Inadequate information infrastructure	53, 61
	Inefficient information system	31, 34, 66
	Poor data accessibility	33, 37, 45, 46, 56, 61, 63
	Low data quality	32, 33, 38, 40, 41, 42, 43, 45, 46, 47, 48, 50, 51, 53, 54, 55, 56, 57, 58, 61, 63, 69, 73
	Record keeping challenges	
	Inconsistency of program and information systems	
**Workforce issues**	Poor workforce engagement	32, 37, 38, 43, 56, 61
	Inadequate skill in data recording	38, 41
	Lack of proper incentives	40, 47
**Evaluation budget**	Insufficient evaluation resource	32, 35, 37, 41, 45, 61, 62
	Limitation of research capacity	
**Evaluation system**	Lack of evaluation plan	31, 62
	Lack of appropriate indicators and tools	

## 4 Discussion

### 4.1 Summary of Findings

This scoping review analyzed 50 studies on NCD program evaluations published between 2006 and 2023, with 76% published post‐2017. Geographically, the majority of studies were from the Eastern Mediterranean Region (24.48%), while the fewest were from the African Region (6.12%). In terms of income levels, most studies were conducted in upper‐middle‐income countries (45.83%), and the least in low‐income countries (4.16%). Regarding study design, 44% were observational, 30% mixed‐methods, 24% interventional, and 2% qualitative. A detailed analysis revealed that 34% employed a cross‐sectional approach. Findings were categorized into three main areas: evaluation processes (including evaluation level, focus, scope, approaches, methods, frameworks, and existing capacities), dimensions and indicators (inputs, processes, outputs, and outcomes), and evaluation challenges (information management, workforce issues, budget constraints, and evaluation systems).

### 4.2 Interpreting of Findings

This study provides key insights into the evaluation of NCDs in PHC, which are discussed below. We found that only 4.16% of evaluation studies were conducted in low‐income countries, highlighting a major research gap. Hategeka et al. (2022) report similar findings [82]. Given that this gap may limit effective NCD interventions and contribute to high mortality in LMICs, strengthening national‐level research capacities should be prioritized, as progress toward a 30% reduction in NCD mortality depends on focused research efforts in this field [3, 83, 84].

Our review showed that the studies used a variety of study designs to evaluate NCD programs, with 44% reporting limitations that affected the certainty and generalizability of results, highlighting the need for careful design selection in future evaluations. appropriate selection of evaluation design—based on implementation stage, setting, resource availability, and stakeholder acceptability—is essential for producing reliable and actionable evidence [85, 86] Three (6%) of studies employed Cluster Randomized Controlled Trials (CRCTs), which are suitable for assessing group‐level interventions [87]. Compared to individual RCTs, CRCTs address contamination risks but require greater logistical coordination [87]. While no study in our sample used standard individual RCTs, they were referenced as the gold standard for causal inference [87], highlighting a gap between ideal and feasible designs that should guide methodological choices in future evaluation. Although no study in our review used a Stepped‐Wedge Cluster Randomized Trial (SW‐CRT) or Interrupted Time Series (ITS) design, these methods were included in the comparative analysis due to their relevance in real‐world program evaluation. SW‐CRTs [87] are especially useful when interventions are expected to be beneficial and cannot be rolled out simultaneously, while ITS designs allow for stronger attribution by tracking trends before and after implementation [87, 88]. Their absence in reviewed studies reflects a potential underuse of designs that balance rigor with ethical and practical considerations in NCD evaluations. Nine (18%) of studies applied quasi‐experimental approaches, mainly pre–post designs. While these are practical in low‐resource or non‐randomized settings, they carry risks of bias due to lack of temporal controls—underscoring the value of more robust alternatives like ITS when feasible [87].

The findings indicate that nonexperimental study designs, including cross‐sectional, cohort, mixed‐methods, and qualitative studies, were widely utilized. Cross‐sectional studies comprised 17 (34%) of the research, providing a quick overview of the intervention results. While these studies offer valuable immediate insights and are relatively easy to conduct in resource‐constrained environments, they are insufficient for fully determining the effectiveness of interventions [87, 89]. In contrast, cohort studies, which were used in five (10%) of the studies, are better suited for evaluating the long‐term impacts of interventions. However, their implementation may be limited by factors such as the study duration, costs, the nature of the intervention, and policy timelines [87]. These patterns underscore the importance of selecting study designs that balance feasibility, rigor, and actionable insights for policy and program planning.

The results indicate that mixed‐method study designs comprised 30% of the investigations, while qualitative studies made up less than 2%. highlighting the limited use of qualitative approaches. Given the chronic nature of NCDs and the complexity of interventions, mixed‐method designs are crucial for examining implementation processes, intervention contexts, components, and stakeholder behaviors [82, 90]. Based on these findings, study design, sample size, sampling methods, and other methodological factors should be aligned with program type, objectives, and evaluation questions, while considering feasibility and stakeholder acceptability, to ensure rigorous and actionable evaluations [87, 91].

Regarding the evaluation process, most studies focused on operational‐level assessments of NCD programs. While these are essential for service delivery, they may not fully reflect overall health system performance. To address this limitation, evaluations should be conducted at multiple levels, including operational centers, communities, and national policy levels. Such multi‐level assessments provide a more holistic perspective, which is crucial for informed policymaking and program redesign [11, 78, 79].

Most evaluations focused on the pilot phase of interventions. While pilot‐phase evaluations are essential for understanding initial implementation, they are not sufficient; evaluations should also include the program's development and scaling‐up stages to provide comprehensive insights for policymakers and enhance program impact [18, 82].

According to our findings, most NCD program evaluations had focused on cardiovascular diseases, diabetes, hypertension, and related risk factors. Achieving SDG Target 3.4 requires comprehensive implementation and evaluation of interventions across all major NCDs, as focusing on only one or two conditions is not sufficient to achieve this target [92]. WHO has suggested a comprehensive NCD package (PEN) for LMICs to strengthen PHC systems [93]; however, many of these countries still focus on cardiovascular diseases and diabetes. Their primary healthcare systems need to be reoriented to comprehensively address all major NCDs, with particular emphasis on cancer and COPD management [93].

Despite the challenges faced by NCD programs, only 4 studies (8%) have evaluated the performance of public–private partnerships (PPPs) in NCD prevention and control. The private sector plays an important role in policymaking, coordination, procurement, financing, health promotion, innovation, and capacity building in NCD management [94]. Failure to evaluate outsourced programs to the private sector, such as PPPs, poses significant challenges to achieving health system goals in NCDs, as it hinders the identification of strengths and weaknesses, the improvement of service quality, sustainable financing, efficiency, and the development of effective interventions [95, 96]. Consequently, this situation may lead to reduced accountability, loss of stakeholder trust, and failure to effectively control NCDs [96]. As a result, policymakers face difficulties in making informed decisions and allocating resources efficiently, which undermines both the effectiveness and sustainability of programs. A key solution is developing integrated databases linking public and private sector data, as limited access and fragmented information impede effective evaluation [96, 97].

Our review identified four distinct evaluation approaches in NCD programs: outcome‑based (48%), process‑based (16%), formative–outcome (used in only one study [77], and process–outcome (34%). The process–outcome hybrid, adopted by over one‑third of studies, emerged as the most comprehensive, capturing both implementation fidelity (e.g., coverage, adherence, and contextual barriers) and program effectiveness (e.g., health outcomes or behavior change). This aligns with updated guidance from the UK Medical Research Council, which advises integrating process and outcome evaluations to illuminate not just if an intervention works, but how and why it does, especially in complex, adaptive systems [98]. Solely outcome‑based approaches risk obscuring critical implementation issues, while process‑only studies fail to demonstrate actual impact in the population. Meanwhile, the rare yet promising formative–outcome approach enables iterative adaptations during rollout while still measuring end‑results, offering a dynamic framework for evolving interventions. Against the multifaceted backdrop of NCD interventions—marked by multi‐component strategies, stakeholder engagement, and contextual variability [99]. We recommend future evaluations adopt a tripartite model combining formative, process, and outcome elements. Such an integrated design supports real‐time course correction, accountability to health outcomes, and deeper insight into causal mechanisms, ultimately enhancing both internal validity and policy relevance.

In terms of data collection methods, most reviewed studies employed quantitative methods (68%), followed by mixed‐methods (30%) and qualitative (2%). This indicates a predominant focus on outcome measurement and causal inference, which quantitative methods are best suited for [87]; Qualitative methods are more useful for formative and process evaluations, especially during early implementation phases, while mixed methods help with data triangulation and understanding context [99, 100]. After deciding on the evaluation design, it is essential to choose data collection methods that align with the evaluation's objectives and research questions [87].

In this review of 15 studies that examined managers/policymakers, service providers, and patient perspectives, the viewpoints of service providers were the most frequently represented (40%). In contrast, patient perspectives appeared in only one study (6.7%), and manager perspectives were included in two studies (13.3%). Comprehensive integration of all three stakeholder groups was observed in only one study (6.7%), while combined patient–provider perspectives were included in five studies (33.3%). This imbalance reflects a predominant emphasis on the operational aspects of service delivery, with less attention given to patient experiences and policy‐level considerations. Such disproportion may limit comprehensive understanding and impact the design and implementation of effective health policies and care programs. Engaging all stakeholders throughout the evaluation process—from design to data collection and analysis—enhances program validity and fosters ownership. Key strategies such as clear communication, regular meetings, inclusive data collection, and co‐creation of evaluation criteria promote transparency and mutual learning, enhancing the outcomes of participatory evaluation [99, 101].

This review revealed the use of diverse evaluation frameworks in NCD program assessments, each addressing specific aspects of program performance. The **Logic Model** [102], was used to map the flow from inputs to outcomes; the **IHHP Model** [79], derived from it, was adapted to local goals, effectively covering various program dimensions. The **Donabedian Model** [103], widely used for assessing healthcare quality, focused on structure, process, and outcomes. One study [30]. expanded this model by integrating the TRIAD framework and cognitive theory to develop a broader theoretical model. The **Kruk Model** [104], went further by emphasizing patient experience, trust, and system responsiveness, in addition to structural and procedural elements—making it especially relevant in low‐resource settings. The **Effective Coverage** [105], framework assessed not only access and quality but also population‐level need, helping to identify service gaps. The **RE‐AIM** [106], model examined interventions through five dimensions—reach, effectiveness, adoption, implementation, and maintenance—providing insight into real‐world impact and long‐term viability. The **PAHO–HEARTS** [107], framework offers standardized core indicators across facility, subnational, and population levels to monitor hypertension control programs. The **CINDI** [108], model applied a multisectoral, population‐based approach to assess inputs, strategies, outputs, and outcomes, generating policy‐relevant evidence. Finally, the **PAST** [109], model offered a structured lens for evaluating program sustainability, focusing on leadership, organizational capacity, partnerships, adaptability, and financing. These findings underscore the value of selecting or combining models based on the evaluation purpose, complexity of the intervention, and contextual factors, to improve the quality, sustainability, and policy relevance of NCD programs. therefore, evaluation frameworks (e.g., IHHP) that allowed contextual flexibility—such as adapting to local systems, data, and stakeholder needs—were more effective in producing meaningful results. This highlights the importance of designing adaptable strategies, particularly for diverse and resource‐limited settings.

Our review highlights significant disparities in research systems across income levels. In LMICs, 86.6% of evaluations were funded internationally and 73% involved international evaluators, reflecting limited domestic capacity and heavy reliance on external support. In contrast, upper‐middle‐ and high‐income countries showed stronger domestic funding (40.9% and 72.7%, respectively) and internal evaluator involvement. These patterns underscore the structural gaps in LMICs' research systems and the need for long‐term investment in local capacity, as emphasized by previous reviews [16, 83]. Furthermore, incomplete reporting of funding sources in several studies calls for improved transparency in research governance.

In terms of **indicators** used, six studies assessed input indicators, including infrastructure, workforce, equipment, medicines, and organizational structures (e.g., care programs and referral systems). These elements are essential for building a robust health system capable of effectively managing NCDs [110]. Inputs evaluation helps to identify the resources available and the resource needed [111]. Findings from the perspectives of service providers, users, and managers highlight that stakeholders often identify critical challenges—such as financing, human resources, equipment, and infrastructure [31, 96]. that underscore the importance of evaluating program inputs in NCD initiatives. In process evaluation of a NCD program, capturing stakeholders' perspectives can help assess the quantity and quality of inputs, and play a significant role in shaping program outcomes, as participatory evaluation enables the identification of weaknesses and the application of effective solutions [99].

In this review, process indicators reported in 16 (32%) of studies. These focus on whether designated activities are performed on time and provide evidence on whether the processes are being followed as intended [111]. These indicators are essential for ensuring effective care delivery, as they reflect actual activities carried out by healthcare providers and patient‐care processes [103]. Despite numerous efforts, significant gaps remain in the detection, diagnosis, and management of NCDs [110]. These persistent gaps highlight the need for thorough process evaluations, therefore, analyzing process metrics, including the roles of providers, recipients, and contextual factors, is critical for improving NCD program effectiveness.

Result indicators were examined in 24 (48%) of studies and included service coverage, effective coverage, patient care and treatment, patient participation, referrals, follow‐up, staff training and mentoring, record keeping, equipped centers, and detection rates. These indicators provide insights into both the quantity and efficiency of services delivered [111]. Inputs indicators are short‐term compared to the outcomes; by measuring them, policy makers can timely implement and monitor interventions, ensuring that health programs remain responsive and effective in achieving their intended goals.

Implementation outcomes—such as feasibility (assessed in 11 studies), and fidelity and adaptation (each assessed in 6 studies)—highlight key aspects of program execution, especially in pilot initiatives. Other outcomes—including reach, dose, sustainability, acceptability, implementation, maintenance, effectiveness, preparedness, and benefit—provide insights into long‐term program performance and resilience [99]. Despite these assessments, low stability and limited preparedness remain significant challenges, and few studies reported sustainability or readiness indices. The sustainability index refers to structures and processes that facilitate the use of resources to maintain and implement evidence‐based interventions [112]. Readiness refers to the availability of necessary capacities for providing services [45]. The COVID‐19 pandemic further highlighted the importance of these metrics, as disruptions in NCD services revealed vulnerabilities in program continuity [113, 114]. These findings emphasize the need for more focused implementation evaluations to strengthen NCD program delivery and long‐term impact.

Other implementation outcomes—such as patient and staff awareness, treatment‐related expenses, physician–patient relationships, service utilization, satisfaction, and healthcare accessibility—were assessed in 11(22%) of studies. Improving these aspects, including provider knowledge, patient health literacy, service affordability, satisfaction, relationships, and accessibility, can enhance service use and contribute to achieving program goals [37]. Therefore, integrating these indicators into evaluations of NCDs is essential for identifying implementation challenges and informing policy decisions.

Clinical outcomes were reported in most studies (38; 76%), underscoring their key role in evaluating NCD programs. These indicators—which varied across the interventions—should be reported at both operational and policy levels, with an emphasis on metrics such as effective coverage, morbidity, and mortality. Adopting standardized outcome measures, aligned with global frameworks such as WHO's NCD Global Monitoring Framework [115], enhances comparability and supports evidence‐based policymaking. Standardization strengthens program design, enables adaptive interventions, and aligns with long‐term health goals including universal health coverage and SDG 3.4 [116].

Several **challenges** in evaluating NCD programs were identified, including weak health information systems, workforce issues, budget limitations, and inadequate evaluation mechanisms.

In terms of information systems, limited data accessibility, poor data quality, inadequate information infrastructure, inefficient systems, and inconsistencies between program and information systems were commonly cited as key obstacles to evaluation in over half of the studies. These shortcomings make conducting evaluations challenging, as reliable assessments depend on the availability of high‐quality data, including completeness, accuracy, timeliness, and coherence [117].

Access to high‑quality baseline data is fundamental to evaluating NCD interventions. Inadequate data access— due to the absence of integrated registries, insufficient data collection protocols, and weak IT infrastructure — leads to selection bias and measurement error. These issues result in under‑ or over‑estimation of intervention effects and insufficient coverage of key clinical outcomes, thereby undermining the certainty and generalizability of findings [30, 66]. Evaluators and researchers working in the field of NCDs, especially in LMICs, can implement practical solutions such as paper‐based or electronic registries, standardized data collection protocols, and validated tools. For example, Moldova's WHO PEN pilot—using adapted guidelines, structured training, and paper records—resulted in sustained improvements in hypertension control and cardiovascular risk detection over 2 years [43, 44]. Digital tools and Artificial Intelligence (AI) can further enhance data quality, reduce errors, and improve the accuracy and timeliness of evaluations.

Human resource–related issues were also reported as another key challenge, including inadequate data collection skills, limited workforce engagement, and lack of proper incentives. Beyond the importance of information systems and data quality in evaluation, active involvement of the health workforce is vital for improving data quality, registries, and other evaluation processes [40]. Insufficient provider engagement and limited data collection skills hinder timely and accurate recording of NCD data and adherence to collection protocols [33]. To address this, practical strategies include supportive supervision, the use of user‑friendly data collection methods, and giving providers ownership in the process. Engaging private‐sector providers through user‐friendly paper registries and supportive on‐site supervision—such as the urban NCD sentinel surveillance in Pune, India—proved effective in enhancing data completeness and timeliness for hypertension and diabetes reporting [118].

Insufficient budget allocation was a key obstacle to evaluation. The evaluation budget should be determined based on the type of program, scope, goals, evaluation design, socioeconomic and organizational context, time frame and method of dissemination of results [24]. Budget constraints can lead to delays in evaluation aligned with intervention phases, reliance on nonexperimental study designs, reduced sample sizes, shorter follow‐up periods, and increased use of secondary data sources [63, 69]. In resource‐limited settings, implementing operational strategies can bridge evaluation gaps and prevent delays. For example, Bhutan's adaptation of the WHO PEN HEARTS program employed intersectoral collaboration, evaluator training, study scope and timeline limitations, and the use of nonexperimental methods and secondary data to enhance NCD management [33].

In terms of evaluation system, Lack of appropriate evaluation plan and indicators and tools were other challenges for evaluation of NCDs. Developing a structured evaluation plan with measurable criteria and selecting appropriate indicators and tools—considering program type, evaluation design, contextual factors, and health system conditions—facilitates effective assessment [31, 62]. Malaysia's enhanced primary healthcare program for NCDs demonstrates the value of planning, frameworks, stakeholder engagement, and measurable indicators [119]. Similarly, WHO PEN HEARTS evaluations in Bhutan [33] and Tajikistan [53] exemplify structured approaches to NCD management in resource‐limited settings.

It is important to note that some included studies exhibited methodological limitations, such as small sample sizes or absence of control groups. These limitations were identified through our thematic analysis and classified under evaluation methods. Using the WHO evaluation process model, we systematically captured potential biases, while the Logic Model guided the identification of indicators across input, process, output, and outcome dimensions. These frameworks clarify how study design and evaluation approaches shape analysis and interpretation, highlighting broader systemic and contextual issues in NCD program evaluation.

### 4.3 Implications for Policy and Practice

The gaps in NCD program evaluations—including limited evaluation of private‐sector initiatives and reliance on observational designs—undermine the evidence base needed for effective decision‐making. Addressing these gaps is critical to generating relevant, context‐specific data that can improve health outcomes and promote equity in service delivery. Strengthening local evaluation capacity, especially in LMICs, is crucial for reducing dependence on external resources and enhancing policy sustainability and impact.

### 4.4 Limitations

To our knowledge, this is the first scoping review to systematically map the technical aspects of NCD program evaluation. However, several limitations should be noted. First, we relied exclusively on publicly available sources and were unable to access the full texts of certain studies, despite contacting authors. Second, gray literature was excluded, which may have omitted relevant findings. Third, this scoping review did not include a formal quality assessment of the included studies, as it was not part of the review's objective. However, this may limit the ability to assess the strength and reliability of the evidence, and findings should therefore be interpreted with caution. Fourth, only studies published in English were included, which may limit the generalizability of findings. Fifth, this scoping review provides a descriptive summary of evaluation methods and challenges in NCD program evaluations; however, its findings may not be directly generalizable to all healthcare systems due to variations in infrastructure, resources, and policies. Readers should interpret the results cautiously, considering contextual differences. Further research is needed to assess the applicability of these findings in specific settings. Sixth, as this was a scoping review, sensitivity analysis was not applicable. However, we acknowledge that methodological variation among studies may influence the interpretation of findings. Finally, we did not perform a formal bias assessment in this scoping review. Potential biases, including publication and selection bias, may influence the findings and should be considered when interpreting the results.

## 5 Conclusion

This scoping review found that evaluations of NCD programs within PHC have predominantly employed observational, cross‐sectional designs. Although these designs offer logistical advantages, they limit causal inference, highlighting the need for greater methodological diversity and rigor. Most evaluations focused on NCD conditions such as cardiovascular diseases (CVDs), diabetes, and hypertension, whereas COPD and cancers received significantly less attention, revealing critical disease‐focus gaps. Evaluations primarily reflected public‐sector programs and the perspectives of service providers, resulting in underrepresentation of other stakeholders. Additionally, in LMICs, most studies were externally funded and conducted by external evaluators, indicating reliance on external expertise and resources. Evaluation indicators were largely focused on outputs and clinical outcomes, while inputs and processes were often overlooked. Furthermore, systemic barriers—including weak information systems, workforce challenges, budget constraints, and inadequate evaluation systems—further limited evaluation rigor. Overall, these findings underscore the urgent need to enhance methodological diversity in NCD program evaluation, address disease‐focus imbalances, incorporate multiple stakeholder perspectives, and strengthen domestic capacities to develop comprehensive and sustainable evaluation frameworks. These insights directly inform the following recommendations.

## Author Contributions

Ali Vafaee‐Najar, Elaheh Houshmand, Masoumeh Sadeghi, Hamidreza Shabanikiya, and Aboalfazl Marvi participated in designing the study. Masoumeh Sadeghi designed the methodology. Masoumeh Sadeghi, PMohsen Aarabi and Aboalfazl Marvi prepared the search strategies. Masoumeh Sadeghi and Aboalfazl Marvi conducted the database search, gathered the data, and screened and selected the studies. Aboalfazl Marvi, Masoumeh Sadeghi, and Mohsen Aarabi conducted the content analysis and evidence synthesis and drafted, reviewed, and prepared the preliminary manuscript. Fatemeh KokabisaghiS, Aboalfazl Marvi, and Masoumeh Sadeghi drafted the English manuscript. Ali Vafaee‐Najar and Elaheh Houshmand supervised the study. All the authors reviewed and approved the final manuscript.

## Funding

The authors received no specific funding for this work.

## Ethics Statement

This study was approved by the Research Ethics Committee of Mashhad University of Medical Sciences (MUMS) (Code: IR.MUMS.FHMPM.REC.1401.156).

## Conflicts of Interest

The authors declare no conflicts of interest.

## Transparency Statement

The lead author Elaheh Houshmand, Ali Vafaee‐Najar affirms that this manuscript is an honest, accurate, and transparent account of the study being reported; that no important aspects of the study have been omitted; and that any discrepancies from the study as planned (and, if relevant, registered) have been explained.

## Supporting information

Revised Supplementary file1 Version 4.

## Data Availability

The data that support the findings of this study are available on request from the corresponding author. The data are not publicly available due to privacy or ethical restrictions.
