# Supplementary material for: A Scoping Review of Noncommunicable Disease Programs Evaluation: Bridging Gaps and Improving Frameworks in Primary Healthcare
Source: Health Sci Rep. 2025 Dec 31;9(1):e71444. doi: 10.1002/hsr2.71444 (PMC12754266; doi:10.1002/hsr2.71444)
Supplement: Supplementary file 1 — Revised Supplementary file1 Version 4. [file HSR2-9-e71444-s001.docx]

**Supplementary file1**

This file is part of the manuscript " **A scoping review of noncommunicable disease programs evaluation: Bridging gaps and improving frameworks in primary healthcare"**

| **Table of content** | | |
| --- | --- | --- |
| **Item** | **Title** | **Page** |
| Appendix1 | The Preferred Reporting Items for Systematic Review and Meta-Analysis extension for scoping review (PRISMA-ScR). | 2-3 |
| Appendix2 | Search strategy terms in selected electronic databases. | 3-4 |
| Appendix3 | The flowchart of coding process | 5 |
| Appendix4 | Diagram of published studies on NCDs evaluation per years | 6 |
| Appendix5 | Inputs, Process and outcome (effectiveness) evaluation indicators | 7-16 |
| Appendix6 | Stakeholders' perspective in process evaluation. | 17-20 |
| Appendix7 | The extraction data form | 21-24 |
| Appendix8 | Coding Definitions Breakdown | 25 |

| **Appendix1: The Preferred Reporting Items for Systematic Review and Meta-Analysis extension for scoping review (PRISMA-ScR) (20).** | | | |
| --- | --- | --- | --- |
|  | **PRISMA ScR Checklist** | | |
| Presented | PRISMA-ScR Checklist Item | Item | **Sections** |
| ✓ | Identify the report as a scoping review. | 1 | **Titel** |
|  |  |  | abstract |
| ✓ | Provide a structured summary that includes (as applicable) background, objectives, eligibility criteria, source of evidence, charting methods, result, and conclusion that related to review questions and objectives. | 2 | Structured summary |
|  |  |  | **Introduction** |
| ✓ | Describe rationale for the review in the context of what is already known. Explain why review questions/objectives lend themselves to a scoping review approach. | 3 | Rationale |
| ✓ | Provide an explicit statement of the questions and objectives being addressed with reference to their key elements (e.g., population or participants, concept, and context) or other relevant key element used to conceptualize the review question and/ or objectives. | 4 | Objectives |
|  |  |  | **Methods** |
| ✓ | Indicate whether a review protocol exist; state if and where can be accessed (e.g., a web address); and if available, provide registration information, including the registration number. | 5 | Protocol and registration |
| ✓ | Specify characteristics of the source of evidence used as eligibility criteria (e.g., years considered. Language, and population status) and provide a rationale. | 6 | Eligibility criteria |
| ✓ | Describe all information sources in the search (e.g., database with dates of coverage and contact with authors to identify additional sources), as well as the data the most recent search was excluded. | 7 | Information sources |
| ✓ | Present the full electronic search strategy for at least 1 database, including any limits used, such that it could be repeated. | 8 | search |
| ✓ | State the process for selecting sources of evidence (i.e., screening and eligibility) included in the scoping review. | 9 | Selection of source of evidence |
| ✓ | Describe the method of charting data from the included sources of evidence (e.g., calibrated forms or forms that have been tested by the team before their use, and whether data charting was done independently or in duplicate) and any process for obtaining and confirm data from investigators. | 10 | Data charting process |
| ✓ | List and define all variables for which data was sought and assumptions and simplifications made. | 11 | Data item |
| - | If done, provide a rational for conducting a critical appraisal of included sources of evidence; describe the methods used and how this information was used in any data synthesis (if appropriate). | 12 | Critical appraisal individual source of evidence |
| - | Not applicable for scoping reviews. | 13 | Summary measure |
| ✓ | Describe the methods of handling and summarizing the data that were charted. | 14 | Synthesis of result |
| - | Not applicable for scoping reviews. | 15 | Risk of bias across studies |
| - | Not applicable for scoping reviews. | 16 | Additional analysis |
|  |  |  | **Result** |
| ✓ | Give numbers sources of evidence screened, assessed for eligibility, and included in the review with reasons for exclusion at each stage, ideally using a flow diagram. | 17 | Selection of source of evidence |
| ✓ | For each source of evidence, present characteristic for which data were charted and provide the citations. | 18 | Characteristic of source of evidence |
|  | If done, present data on critical appraisal of included sources of evidence (see item 12). | 19 | Critical appraisal within source of evidence |
| ✓ | For each included source of evidence, present the relevant data that were charted that relate to the review questions and objectives. | 20 | Result of individual source of evidence |
| ✓ | Summarize and/ or present the charting results as they related to the review questions and objectives. | 21 | Synthesis of result |
| - | Not applicable for scoping reviews. | 22 | Risk of bias across studies |
| - | Not applicable for scoping reviews. | 23 | Additional analysis |
|  |  |  | **Discussion** |
| ✓ | Summarize the main results (including an overview of concepts, themes, and type of evidence available) link to the questions and objectives, and consider the relevance to key groups. | 24 | Summary of evidence |
|  | Discuss the limitation of the scoping review process. | 25 | Limitation |
| ✓ | Provide a general interpretation of the results with respect to review questions and objectives.as well as potential implication and /or next steps. | 26 | **Conclusions** |
| ✓ | Describe the source of funding for the included sources of evidence, as well as sources of funding for the scoping review, describe the role of the funders of the scoping review. | 27 | **Funding** |

| **Appendix2: the search strategy** | | |
| --- | --- | --- |
| **results** |  | **Database/electronic journal/ website** |
| 5883 | **((((((((((((((((((("Noncommunicable Diseases"[Mesh]) ) OR ("noncommunicable diseases"[All Fields])) OR ("noncommunicable disease"[All Fields])) OR ("noncommunicable"[All Fields])) AND ("diseases"[All Fields])) OR ("noncommunicable"[All Fields])) AND ("disease"[All Fields])) OR ("non communicable diseases"[All Fields])) OR ("Non-communicable Diseases"[All Fields])) OR ("Non-communicable Disease"[All Fields])) OR ("non communicable diseases"[All Fields])) OR ("chronic disease"[All Fields])) OR ("chronic diseases"[All Fields])) OR ("noncommunicable diseases prevention and control"[All Fields])) OR ((((("Non-infectious Disease"[All Fields]) OR ("Non-infectious Diseases"[All Fields])) OR ("non infectious diseases"[All Fields])) OR ("noninfectious diseases"[All Fields])) OR ("noninfectious disease"[All Fields])))) AND (((((((((((((((((((((((("evaluation"[All Fields]) OR ("evaluation model"[All Fields])) OR ("evaluation models"[All Fields])) OR ("evaluation framework"[All Fields])) OR ("evaluation framework design"[All Fields])) OR ("health care evaluation"[All Fields])) OR ("health services evaluation"[All Fields])) OR ("program evaluation"[All Fields])) OR ("program evaluation model"[All Fields])) OR ("program evaluation framework"[All Fields])) OR ("process evaluation"[All Fields])) OR ("assessment"[All Fields])) OR ("monitoring"[All Fields])) OR ("program monitoring"[All Fields])) OR ("management"[All Fields])) OR ("intervention and evaluation"[All Fields])) ) OR ("implementation and evaluation"[All Fields])) OR ("analysis and evaluation"[All Fields]))) OR (((("surveillance"[All Fields]) OR ("surveillance system"[All Fields])) OR (surveillance evaluation)) OR ("surveillance monitoring"[All Fields]))) OR ("health care evaluation mechanisms"[All Fields])))) OR ("Cost-Effectiveness Analysis"[Mesh]))) AND ((("Primary Health Care"[Mesh]) OR ("primary health care"[All Fields])))** | PubMed Database |
| 6960 | ( "non communicable diseases" OR "non communicable diseases prevention and control" OR "package of essential non communicable diseases intervention" ) AND ( "primary health care" OR "PHC" ) AND ( ( "evaluation model" OR "evaluation framework" OR "evaluation framework design" OR "health care evaluation" OR "health services evaluation" OR "program evaluation" OR "program evaluation framework" OR "process evaluation" OR "assessment" OR "monitoring" OR "program monitoring" OR "management" OR "intervention and evaluation" OR "implementation and evaluation" OR "analysis and evaluation" OR "surveillance system" OR " cost effectiveness analysis" ) OR ( performance ) ) | Scopus Database |
| 9586 | **((((((((((TS=(Noncommunicable Diseases)) OR TS=(Noncommunicable Disease)) OR TS=(Non-communicable Diseases)) OR TS=(Non-communicable Disease)) OR TS=(chronic disease)) OR TS=(chronic diseases)) OR TS=(noncommunicable diseases prevention and control)) OR TS=(Non-infectious Disease)) OR TS=(Non-infectious Diseases)) OR TS=(noninfectious disease)) OR TS=(noninfectious diseases) AND (((((((((((((((((((TS=(evaluation models)) OR TS=(evaluation)) OR TS=(evaluation model)) OR TS=(evaluation framework)) OR TS=(evaluation framework design)) OR TS=(health care evaluation)) OR TS=(health services evaluation)) OR TS=(program evaluation)) OR TS=(program evaluation model)) OR TS=(program evaluation framework)) OR TS=(process evaluation)) OR TS=(assessment)) OR TS=(monitoring)) OR TS=(program monitoring)) OR TS=(management)) OR TS=(intervention and evaluation)) OR TS=(implementation and evaluation)) OR TS=(surveillance system)) OR TS=(analysis and evaluation)) OR TS=(cost effective analysis) AND (TS=(primary health care)) OR TS=(PHC) AND TS=(performance)** | Web of science Database |
| 10787 | (('primary health care'/exp OR 'primary health care') AND ('non communicable disease'/exp OR 'non communicable disease' OR 'chronic disease'/exp OR 'chronic disease') AND (('performance'/exp OR 'performance') OR ('evaluation study'/exp OR 'evaluation study' OR 'evaluation and follow up'/exp OR 'evaluation and follow up' OR 'monitoring'/exp OR 'monitoring' OR 'management'/exp OR 'management' OR 'disease management'/exp OR 'disease management' OR 'cost effectiveness analysis'/exp OR 'cost effectiveness analysis' OR 'model'/exp OR 'model' OR 'analysis'/exp OR 'analysis') OR ('outcome assessment'/exp OR 'outcome assessment' OR 'health care quality'/exp OR 'health care quality' OR 'process design'/exp OR 'process design' OR 'health care facilities and services'/exp OR 'health care facilities and services')) | Embase Database |
| 1184 | "Non communicable diseases" OR "non-communicable diseases" OR "chronic diseases" AND (evaluation OR "Evaluation model" OR framework OR design OR process OR "follow up" OR "intervention and evaluation" OR program OR "program Monitoring" OR "cost-effectiveness" OR "Health care services" OR Management OR performance OR "evaluation method" OR intervention OR measurement OR analysis OR "outcome assessment" OR "pilot program" OR surveillance) AND ("Primary health care" OR PHC) | Emerald electronic journal |
| 9 | Noncommunicable diseases AND Evaluation AND PHC OR Primary health care)  Diabetes care evaluation, Diabetes services evaluation, hypertension program evaluation, Diabetes program evaluation, cancer screening evaluation, chronic diseases evaluation | Google Scholar |
| 0 | Hand searches: Noncommunicable diseases program evaluation, NCDs evaluation, NCDs evaluation in primary health care | WHO,  CDC,  World bank website |
| 3 | - | Bibliography of the included studies |
| 34412 |  | Final |

**Appendix3: The flowchart of coding process**

Review and confirm the results

Reviewing of extracted data and results to ensure that the data is organized correctly.

Finally, the research team reviewed the coding process and confirmed the result

Review and confirm the results

Reviewing of extracted data and results to ensure that the data is organized correctly.

Finally, the research team reviewed the coding process and confirmed the result

Extraction and categorizing

Extracting relevance data, and categorizing the data according to predefined codes. In conclusion final themes emerged.

Extraction and categorizing

Extracting and categorizing the primary codes, condensing them, and grouped into subcategories based on similarities and differences in concept. Finaly the main themes revealed.

data- driven codes/ Open coding

Reading sources of evidence verbatim, extracting relevance data using open coding (word, meaning unit).

Predefined codes

Primary codes were determined by extracting domains and sub-domains from the chosen theoretical frameworks. reading the texts of evidence sources word by word and extracting relevance data.

Immersion in data

Reviewing and reading Sources of evidence at least three times to ensure full familiarity and comprehension of the subject.

Inductive coding approach

Deductive coding approach

**Appendix4: diagram of published studies on NCDs evaluation per years**

Appendix5: Process and outcome (effectiveness) evaluation indicators

| **Study references** | **Inputs indicators** | **Process indicators** | **Outputs** | **Clinical outcomes** | **other outcomes** | **implementation outcome** |
| --- | --- | --- | --- | --- | --- | --- |
| 30 |  | 1.Lab investigation  2.Clinical assessment  3.Patients Counselling  4.Medicine prescription  5.Staff training | - | 1.HbA1c control  2.LDL control  3.HDL control  4.BP control* | - | - |
| 31 |  | - | - | - | - | Sustainability  1.Environment supports  2. partnership  3. financing  4. Evaluation  5. organization  6. adaptation  7. communication  8.strategic plan  9. strengths and challenges |
| 32 |  | 1.Cancer Screening | 1.Education rate  2.Screenining rate  3.Number of visits | 1.Detected rate  2.Patients adherence | - | 1.Feasibility  2.Effectiveness |
| 33 | -Human resources availability accessibility  -Availability of basic devices  - Facility with presence of care delivery and referral systems  - Medicines and diagnostic supplies availability  -Access to essential medications and diagnostic tools | - | 1.Staff trained  2.Staff Mentoring  3.Staff supervision  4.Social media use  5.Center with essential medicine and tools  6.Patient – centered care  7.Record keeping | 1.BP control  2.FBS control  3.Treatment gap  4.Patients adherence  5.Patient trained | 1.Staff awareness | 1.Strengths  2. limitation |
| 34 |  | - | - | 1.Incidence rate of Myocardial infraction &stroke  2.Myocardial Infraction Mortality Rate  3.Stroke Mortality Rate | - | - |
| 35 |  | - | - | 1.Health Behavior change  2.Changing at SBP  3.Changing at DBP  4.Change at HbA1c  5.Changing at FBS  6.Change at LDL level | 1.Motivation and recognition  2.Doctor -patient’s relationship  3.Medical Services Utilization  4.Practice pattern change in  Health provider | - |
| 36 |  | - | - | 1Complication  2.FBS control  3.HbA1c control  4.Lipid profile control  5.BP, HbA1c, LDL control | 1.Services utilization  2.Access to health care | - |
| 37 |  | - | - | 1.Changing at SBP  2.Changing at DBP  3.Changing health behaviors | 1.Diseases recognition  2.Motivation for change behavior  3.Regular visit and compliance  4.Physician–patient relationship  5.Medical services utilization | - |
| 38 |  | - | - | - |  | Sustainability:  1.Environment supports  2. partnership  3. financing  4. Evaluation  5. organization  6. adaptation  7. communication  8.strategic plan |
| 39 |  |  |  |  |  |  |
| 40 |  | - | 1.Diagnosis rate  2.Participant rate  3.Referral rate | - | - | - |
| 41 |  | - | - | - | - | 1.Fidelity  2.Feasibility  3.Adaptation  4.Benefit  5. Challenges |
| 42 |  | - | - | 1.HbA1c control  2.BMI index  3.Chronic complication | - | - |
| 43 |  | 1.Medicine prescription  2.Physical examination  3.HbA1c measured  4.Documentation rate  5.CVD risk score assessment  6.Risk factor assessment  7.Staff training | - | 1.BP at control range  2.BP at normal range  3.Patients with elevated BP  4.Life Style Change | - | 1.Feasibility  2.Adaptation |
| 44 |  | 1.Medicine Prescription  2.Physical exam amination  3.HbA1c measured  4.Records keeping | - | 1.BP at control range  2.BP at normal range | - | - |
| 45 | Human resources availability accessibility  -Availability of basic devices  - Medicines and diagnostic supplies availability  -Access to essential medications and diagnostic tools | - | 1.Total screened rate  2.Risk factors detected rate  3.Diabetes detected rate  4.Hypertension detected rate  5.CVD risk assessment rate  6.Suspect cervix cancer detected rate  467Suspect breast cancer detected rate  8.Referral rate in suspected cancer cases  9.Staff trained rate  10.Humane resource availability | 1.BP control  2.FBS Control  3.CVD risk score Reduction | - | 1.Feasibility  2.Fidelity  3.Facilitators  4.Barriers  5.Health system  6. preparedness |
| 46 |  | - | 1.Treatment rate  2.Coverage rate  3.Patients treatment according the guideline  5.CVD risk assessment | 1.BP control in patients  2.BP control in population | - | - |
| 47 |  | - | 1.Diabetes detected rate  2.Hypertension detected rate | 1.BP control in patients treated  2.FBS Control in patients treated | - | 1.Strengths  2.Challenge |
| 48 |  | - |  | 1.Survival rate  2.Cardiovascular incidence  3.Adherence to treatment | - | - |
| 49 |  | - | - | 1.Mortality rate  2.Complication rate  3.Survival rate  4.Hospitalization rate |  |  |
| 50 |  | 1.Counseling rate  2.Screening rate  3.Medicine Prescribing  4.Physical examination  5.lab investigation rate  6.Records keeping  7.CVD risk assessment | - | 1.BP control in treated patients | - | - |
| 51 |  | - | - | 1.Lipid profile change  2.SBP control  3.DBP control  4.FBS control  5.Complication prevalence  6. HbA1c change  527.BMI change | - | - |
| 52 |  | - | - | 1. Lifestyle change  2.BMI change  3.Quality of life change  4.SBP control  5.DBP control  6.FBS control  7.Lipid Profile Change  8.HbA1c change | - | - |
| 53 |  | 1.Medication prescribing  2.Physical examination  3.lab investigation  4.Risk factors assessment  5.Smoking status recorded  6.CVD risk factors assessment  7.CVD risk score document | 1.Risk factor assessment rate  2.Screening rate  3.Hypertension detected rate  4.Diabetes detected rate  5.Staff trained rate | 1.BP control in normal range  2.BP control in low risks  3.BP control in high risks | - | 1.Feasibility  2.Adaptation |
| 54 | Human resources availability accessibility  -Availability of basic devices  - Medicines and diagnostic supplies availability  -Access to essential medications and diagnostic tools | 1.Physical examination  2.lab investigation | 1. Staff trained rate  2. Centers with:  3.Basic technology  4.Diagnostic equipment’s  5Essential medicine | 1.FBS control  2.BP control  3.Chronic complications:  4.Eye complication  5.Foot complication  6.Neuropathy | No | 1.Patients experiences |
| 55 |  | - | 1.Treatment rate  2.Staff trained rate  3.Referral rate  4.Follow-up rate | 1.SBP control  2.DBP control | 1.Patients' awareness | - |
| 56 |  | - | 1.Screening rate | - | 1. Patients satisfaction | 1.Feasibility  2.Acceptability  3. strengths  4.challenges |
| 57 |  | 1.Screening rate | 1.Detected rate  2.number of cares | 1.Hypertension prevalence | 1.Expenditure on NCDs | 1.Preparedness  2.Patients experiences |
| 58 | Human resources availability accessibility  -Availability of basic devices  - Medicines and diagnostic supplies availability  -Access to essential medications and diagnostic tools | - | -participation rate | 1.Asthma symptom  2.asthma diagnosis  3. quality of life | 1.Patients satisfaction | 1.patients experiences  2.strengths and limitation  3.preparedness |
| 59 |  | - | - | - | - | 1.Fidelity  2.Feasibility  3.Adaptation  4.Accessibility  5.Health provider experience  6.Patients experience |
| 60 |  | - | - | - | - | 1.Fidelity  2.Feasibility  3.Accessibility  4.Health provider experience  5.Patients experience |
| 61 |  | 1.Diseases screening  2.Patients referral  3.Hypertension follow up  4.Diabetes follow up  5.Patients record | - | 1.BP control  2.FBS control | - | 1.Fidelity  2.Dose |
| 62 | - Medicines and diagnostic supplies availability  -Access to essential medications and diagnostic tools | 1.Hypertension screening  2.Hypertension diagnosis  3.Hypertension treatment  4.Hypertension visited record  5.Follow up rate | - | 1.BP control | - | 1.Strengths  2.Weakness |
| 63 |  | 1.CVD risk assessment  2.Medication prescribing  3.Physical examination  4.Lab investigation  5.Documentation  6.Referral rate  7.Follow up rate | 1.Staff trained | 1.BP control  2.FBS control  3.Blood cholesterol  4.CVD risk control  5.BMI change  6.Waist circumference  7.Urine albumin | - | 1.Feasibility |
| 64 | Human resources availability accessibility  -Availability of basic devices  - Facility with presence of care delivery and referral systems  - Medicines and diagnostic supplies availability  -Access to essential medications and diagnostic tools  -Facility availability | 1.Counseling for life style change  2.Guidance for insulin use  3.Lab assessment:  4.HbA1c, FBG, Urine analysis, lipid profile, creatinine  5.Physical examination  6.Complication screening | - | 1.SPB control  2.DBP control  3.FBS control  4.HbA1c control | - | 1.Health provider experience |
| 65 |  | 1.Patients training  2.Staff training  3.Patient follow up | - | 1.Quality-of life  2.COPD control rate  3.Self efficacy score  4.Exercise capacity  5.Preventive measures  -Symptom exacerbation  -Hospitalization rate | - | 1.Feasibility  2.Reach  3.Dosage  4.Fidelity  5.Acceptability  6.Patient and provider feedback |
| 66 |  | - | 1.Hypertension detected rate  2.Diabetes detected rate  3.CVD risk assessment  4.Patients record rate  5.Risk factor screening  6.Referral rate  7.Costs rate  8.Staff trained | 1.Blood pressure high risk visit  2.Myocardial infarction rate  3.stroke rate  4.Cardiovascular death  5.BP control  6.Hospital admissions  7.Ambulance call for hypertensions  8.Number of referrals to hospital | 1.Staff awareness | - |
| 67 |  | - | - | - | - | 1.Strengths  2.Weakness |
| 68 |  | 1.Medication prescribing  2.Physical exam  3.Lab investigation  4.Documentation  5.Patients Referral | 1.Coverage rate  2.Hypertension detected rate  3.Diabetes detected rate  4.renal disease detected rate  5.Risk factors screening rate  696.Treatment rate  7.Physical exam rate  8.Lab investigation rate  9. Documentation rate  10.Referral rate | 1.Raised SPB  2.Raised DBP  3.Raised FBS  4.BMI Control | No | No |
| 69 |  | - | 1.Brest cancer screening rate  2.Detected rate | - | - | - |
| 70 |  | - | Coverage rate  Diabetes screening rate  Hypertension screening rate  Risk factors screening rate | - | - | - |
| 71 |  | - | - | 1.SBP control  2.DBP control  3.Lifestyle change  4.Risk factor change | 1.Hospitalization cost  2.Out patient’s medical cost  3.Medicine cost  4.Patients awareness  5.Services utilization | - |
| 72 |  | - | 1.Staff trained  2.Patients group trained  3.Coverage rate  4.Patients care rate | 1.BP control  2.FBS control  3.LDL control  4.Microalbuminuria control  5.Patients adherence rate | - | 1.Reach  2.Effectiveness 3.Adoption  4.Implementation  5.Maintenance |
| 73 |  | - | 1.Treatment rate | 1.SBP control  2.DBP control  3.FBS control | - | - |
| 74 |  | - | 1.Screening rate  2.Treatment rate  3.Referral rate  4.Follow up rate  5.Referral feedback rate  6.CVD risk assessment  7.Diabetes detected rate  8.Hypertension detected rate  9.Services documentation | 1.CVD risk score change  2.Health behavior improvement  3.BMI change  4.Waist circumference change  5.Medication intake change  6.BP control  7.FBS control  8.Peak expiratory flow change | - | - |
| 75 |  | - | 1.Treatment coverage  2.BP Treatment rate  3.Diabetes treatment rate | 1.Diabetes prevalence  2.Hypertension prevalence  3.SBP control  4.FBS control | - | - |
| 76 |  | 1.Participation rate  2.HbA1C availability  3.FBS availability | Effective coverage:  1.Detected rate  2.Lipid profile  3.Fasting plasma glucose  4.Education rate  5.Hypertension control  6.Complication control | - | - | - |
| 77 |  | - | Number:  1.Participation  2.Coverage  3.Community trained | 1.Behavior risk factor improvement  2.SBP control  3.DBP control  4.Raised BP  5.Overweight prevalence  6.BP monitored  7.FBS monitored | - | 1.Reach  2.Feasibility |
| 78 |  | SBP monitoring  2.DBP monitoring  3.Lifestyle monitoring  4.Risk factor monitoring  5. HbA1c monitoring | - | 1.SBP control  2.DBP control  3.Lifestyle change  4.Risk factor change  5. HbA1c control | - | - |
| 79 |  | - | 1.Number of staff trained  2.Number of patients educated | 1.Patients life style improvement  2.Health Providers  life style change | 1.Health worker’s knowledge improvement  2.Health worker’s activity for patient’s care | - |
|  |  | Abbreviations:  Fasting Blood Saugers (**FBS**), Blood Pressure (**BP**), Systolic Blood Pressure (**SBP**), Diastolic Blood Pressure (**DBP**), Body Mass Index (**BMI**), Low Density Lipoprotein (**LDL**), Cardio Vesicular Diseases (**CVD**), Coronary Obstructive Pulmonary Diseases (**COPD**). | | | | |

**Appendix6: Stakeholders' perspective in process evaluation.**

| **Study references** | **Patients' perspective** | | **Health workers perspective** | | **Health Managers perspective** | |
| --- | --- | --- | --- | --- | --- | --- |
|  | **Facilitators** | **Challenges** | **Facilitators** | **Challenges** | **Facilitators** | **Challenges** |
| Partovi, Y, 2023 (31) | - | - | - | - | - | -Financial  instability, lack of budget allocation  -Evaluation mechanisms -inadequate Resources -deficiencies in intersectoral cooperation  -Failure to utilize of community capacities |
| Tenzin K, 2022 (33) | -Diseases management  -Patients Follow up  -Health provider communication | -Staff shortage  -Waiting time  -Lack of tools | -Support supervision  -Coaching and mentoring  -Performance feedback  -Satisfaction | -Human resource shortage  -Medication shortage  -Equipment failure  -Record keeping  -Insufficient time for patients care  -Process of requesting medicine | - | - |
| Shanmuganathan S.2022(38) | - | - | - | - | Communication  Organization capacity | Funding  Human resource  Staff training  Lack of office space  Inadequate process evaluation  Celerity of responsibility |
| Low LL, 2021(41) | - | - | -Care coordination  -Screening  -Staff Training  - system for referral | -Lacked of building infrastructure  -Staff shortage  -Record keeping  -Referral guideline  -Challenge in services level communication  -Referral guideline  -Waiting time  -Access to internet  -Patients adherence  -Patients follow up | - | - |
| Aye, L. L. et al. 2020(45) | - | - | -Improving human resource availability  -Improving medicine availability  - training of staff | -Programmatic challenges  -Lack of human resources  -Management of hypertension  -Management of diabetes | - | - |
| Flor L. S, 2020 (47) | -Services delivery | -Waiting time | -Enhancing health team communication  -Patients – doctor’s communication  -Process of services delivery  -Patients awareness | -Electronic health record  -Staff shortage  -Equipment’s shortage  -Medicine shortage  -Physical accessibility  -Referral process | -Time saving of services delivery  -Services affordability | - |
| Birabwa, C. et al. 2019(54) | - | - | - | -Equipment’s shortage  -Medicine shortage  -Training and Guideline  -High-cost medicine  -Waiting time  -lack of transport  -Time of accessibility  -Few health worker  -Lack of laboratory equipment's  - clinic take long to open  -sharing pharmacy with other patients  -poor skill of health workers | - | - |
| Peiris, D, 2019(56) | - | - | -Improving relationship | -Insufficient staff training  -services delivery challenges  - sustainability of resource | -- | - |
| Jayanna, K, 2019(57) | - | -Fear, cultural beliefs, and stigma  -High Out‑of‑pocket costs and inadequate public system  Poor patient–provider communication and social support gaps | - | - | - | - |
| Kadhim Yasir, M, 2018(58) | -Satisfaction assessed | - | -Human resource availability  -Equipment availability | -Record keeping  -Services delivery | - | - |
| Yan, L. D. et al. 2017(62) | - | - | - | -Equipment and personnel shortage  - Medication stock-outs  - Poor patient visit attendance | - | - |
| Khan, M. A. et al. 2018(59) | -Patients records  -Advice by documents | -Laboratory accessibility | -Private clinic engagement  -Prescribing  -Record keeping  -Screening | -Staff training  -Limitation building infrastructure  -Patients expectation  -Patients adherence  -Cultural challenge | - | - |
| Khan, M. A. et al. 2018(60) | -Adherence support | -Cultural and social barriers  -Short Intervention duration | -Feasibility in routine settings: skill-based training, structured screening, diagnostic testing, and standardized case management | -Operational challenges: needs for stable drug supply, ongoing supervision, and in-service training to maintain intervention quality |  |  |
| Tania, C. et al. 2017(65) | -Knowledge improvement  -diseases management improvement | -Engagement external provider  -Unclear of program goal  -Organizational challenge  -Lack of incentive | - Focus on healthy life style  - Peer support  - Improved self-efficacy  - Action plan for acute COPD exacerbations | - Lack of information about the programme  -Data sharing  -Patient monitoring  -Services delivery | - | - |
| Collins. D, et al. 2017(67) |  |  | -Mainstreaming of PEN protocols  - Use of lifestyle interventions  - Training for PEN protocols | -Lack of training in risk  Stratification  -Lack of technology to make risk  Prediction  -Lack of information  campaigns for the population  -Lack of educational materials  for use with patients  - Lack of human resources |  |  |

**Appendix7: the extraction data form (part1)**

| Subject# | Author | year | Country of research | Country by WHO regions | Country by World bank income level | Study title | Study design | Study specific setting  (facility level, hospital,) | Study participant/ number of participants | Level of study (city, region, state, national | Title of program/ intervention | Type of program/ intervention (pilot, or program  (full- scale) | Evaluation approach |
| --- | --- | --- | --- | --- | --- | --- | --- | --- | --- | --- | --- | --- | --- |
| #1 | Birabwa, C. et al. | 2019 | Uganda | AFRO | Low-income country | Quality and barriers of outpatient diabetes  care in rural health facilities in Uganda – a  mixed methods study | Mixed methods | Hospitals and Rural Health Centers | 377 diabetes patients in quantitative phase and 8 health providers in qualitative phase including, 2 medical doctors, 2 nurses, and 2 clinical officers. | (district, Micro level) | Diabetes | Program full-scale, | Process evaluation and outcome evaluation |
| #2 |  |  |  |  |  |  |  |  |  |  |  |  |  |
| #3 |  |  |  |  |  |  |  |  |  |  |  |  |  |
| #4 |  |  |  |  |  |  |  |  |  |  |  |  |  |
| #5 |  |  |  |  |  |  |  |  |  |  |  |  |  |
| #6 |  |  |  |  |  |  |  |  |  |  |  |  |  |
| #7 |  |  |  |  |  |  |  |  |  |  |  |  |  |
| #8 |  |  |  |  |  |  |  |  |  |  |  |  |  |
| #9 |  |  |  |  |  |  |  |  |  |  |  |  |  |
| #10 |  |  |  |  |  |  |  |  |  |  |  |  |  |
| #11 |  |  |  |  |  |  |  |  |  |  |  |  |  |
| #12 |  |  |  |  |  |  |  |  |  |  |  |  |  |
| #13 |  |  |  |  |  |  |  |  |  |  |  |  |  |
| #14 |  |  |  |  |  |  |  |  |  |  |  |  |  |
| #15 |  |  |  |  |  |  |  |  |  |  |  |  |  |
| #16 |  |  |  |  |  |  |  |  |  |  |  |  |  |
| #17 |  |  |  |  |  |  |  |  |  |  |  |  |  |
| #18 |  |  |  |  |  |  |  |  |  |  |  |  |  |
| #19 |  |  |  |  |  |  |  |  |  |  |  |  |  |
| #20 |  |  |  |  |  |  |  |  |  |  |  |  |  |
| #21 |  |  |  |  |  |  |  |  |  |  |  |  |  |
| #22 |  |  |  |  |  |  |  |  |  |  |  |  |  |
| #23 |  |  |  |  |  |  |  |  |  |  |  |  |  |
| #24 |  |  |  |  |  |  |  |  |  |  |  |  |  |
| #25 |  |  |  |  |  |  |  |  |  |  |  |  |  |
| #26 |  |  |  |  |  |  |  |  |  |  |  |  |  |
| #27 |  |  |  |  |  |  |  |  |  |  |  |  |  |
| #28 |  |  |  |  |  |  |  |  |  |  |  |  |  |
| #29 |  |  |  |  |  |  |  |  |  |  |  |  |  |
| #30 |  |  |  |  |  |  |  |  |  |  |  |  |  |
| #31 |  |  |  |  |  |  |  |  |  |  |  |  |  |
| #32 |  |  |  |  |  |  |  |  |  |  |  |  |  |
| #33 |  |  |  |  |  |  |  |  |  |  |  |  |  |
| #34 |  |  |  |  |  |  |  |  |  |  |  |  |  |
| #35 |  |  |  |  |  |  |  |  |  |  |  |  |  |
| #36 |  |  |  |  |  |  |  |  |  |  |  |  |  |
| #37 |  |  |  |  |  |  |  |  |  |  |  |  |  |
| #38 |  |  |  |  |  |  |  |  |  |  |  |  |  |
| #39 |  |  |  |  |  |  |  |  |  |  |  |  |  |
| #40 |  |  |  |  |  |  |  |  |  |  |  |  |  |
| #41 |  |  |  |  |  |  |  |  |  |  |  |  |  |
| #42 |  |  |  |  |  |  |  |  |  |  |  |  |  |
| #43 |  |  |  |  |  |  |  |  |  |  |  |  |  |
| #44 |  |  |  |  |  |  |  |  |  |  |  |  |  |

**Appendix7: the extraction data form (part2)**

| Subject# | Methods of evaluation (quantitative / qualitative, mixed methods | Model/Framework/tools used for evaluation | Quantitative indicators based on study finding (inputs, process, out puts, clinical outcomes, other outcome implementation outcomes) | Qualitative themes and subthemes/ perspectives/ challenges based on study finding | Limitation of study or research | Evaluation capacities funding/ (evaluators) | Evaluating challenges |
| --- | --- | --- | --- | --- | --- | --- | --- |
| #1 | Mixed methods | Kruk model | Inputs: staff training, basic technology, diagnosis test, and essential medicine.  Process: blood glucose, blood pressure, eye exam, foot exam, urine protein, and cholesterol. Outcome: blood glucose control, blood pressure control, chronic complication, eye lesion, peripheral nephropathy, and foot lesions | -Equipment’s shortage  -Medicine shortage  -Training and Guideline  -High-cost medicine  -Waiting time  -lack of transport  -Time of accessibility  -Few health worker  -Lack of laboratory equipment's  - clinic take long to open  -sharing pharmacy with other patients  -poor skill of health workers | The use of self-reports which may have resulted in over- or  under- estimation of some indicators.  The assessment of BP control based on a single set of  BP readings may have resulted in under or over estimation of burden of high BP | Internal funder  (Internal evaluators) | Record keeping in patients.  Shortage of document system. |
| #2 |  |  |  |  |  |  |  |
| #3 |  |  |  |  |  |  |  |
| #4 |  |  |  |  |  |  |  |
| #5 |  |  |  |  |  |  |  |
| #6 |  |  |  |  |  |  |  |
| #7 |  |  |  |  |  |  |  |
| #8 |  |  |  |  |  |  |  |
| #9 |  |  |  |  |  |  |  |
| #10 |  |  |  |  |  |  |  |
| #11 |  |  |  |  |  |  |  |
| #12 |  |  |  |  |  |  |  |
| #13 |  |  |  |  |  |  |  |
| #14 |  |  |  |  |  |  |  |
| #15 |  |  |  |  |  |  |  |
| #16 |  |  |  |  |  |  |  |
| #17 |  |  |  |  |  |  |  |
| #18 |  |  |  |  |  |  |  |
| #19 |  |  |  |  |  |  |  |
| #20 |  |  |  |  |  |  |  |
| #21 |  |  |  |  |  |  |  |
| #22 |  |  |  |  |  |  |  |
| #23 |  |  |  |  |  |  |  |
| #24 |  |  |  |  |  |  |  |
| #25 |  |  |  |  |  |  |  |
| #26 |  |  |  |  |  |  |  |
| #27 |  |  |  |  |  |  |  |
| #28 |  |  |  |  |  |  |  |
| #29 |  |  |  |  |  |  |  |
| #30 |  |  |  |  |  |  |  |
| #31 |  |  |  |  |  |  |  |
| #32 |  |  |  |  |  |  |  |
| #33 |  |  |  |  |  |  |  |
| #34 |  |  |  |  |  |  |  |
| #35 |  |  |  |  |  |  |  |
| #36 |  |  |  |  |  |  |  |
| #37 |  |  |  |  |  |  |  |
| #38 |  |  |  |  |  |  |  |
| #39 |  |  |  |  |  |  |  |
| #40 |  |  |  |  |  |  |  |
| #41 |  |  |  |  |  |  |  |
| #42 |  |  |  |  |  |  |  |
| #43 |  |  |  |  |  |  |  |
| #44 |  |  |  |  |  |  |  |

Appendix8: Coding Definitions Breakdown

| Theme | Subtheme | Coding Definition |
| --- | --- | --- |
| Information Management | Inadequate information infrastructure | Lack of sufficient infrastructure to support effective data collection and management. |
|  | Inefficient information system | Systems used for managing information are slow, unreliable, or not user-friendly. |
|  | Poor data accessibility | Difficulty in accessing required data when needed. |
|  | Low data quality | Data contains errors, inconsistencies, or missing values that reduce its reliability. |
|  | Record keeping challenges | Problems related to accurate and consistent documentation of program activities. |
|  | Inconsistency of program and information systems | Mismatch between program data needs and the information systems in place. |
| Workforce Issues | Poor workforce engagement | Lack of motivation, participation, or commitment of staff involved in evaluation activities. |
|  | Inadequate skill in data recording | Insufficient training or expertise in accurately recording data. |
|  | Lack of proper incentives | Absence of rewards or motivation mechanisms for staff to participate effectively in evaluation. |
| Evaluation Budget | Insufficient evaluation resource | Limited financial resources allocated for evaluation activities. |
|  | Limitation of research capacity | Lack of trained personnel and institutional support for conducting evaluations. |
| Evaluation System | Lack of evaluation plan | Absence of a clear, structured plan guiding evaluation processes. |
|  | Lack of appropriate indicators and tools | Missing or inadequate tools and indicators needed to assess program performance effectively. |
